# Optimal ratio or historical convention: the use of methanol–ethanol mixtures as pressure-transmitting mediums

**DOI:** 10.1107/S1600576725008349

**Published:** 2025-10-24

**Authors:** Cameron J. G. Wilson, Cecilia M. S. Alvares, Anna Herlihy, Nicholas P. Funnell, Gabriele C. Sosso, Mark S. Senn

**Affiliations:** a Department of Chemistry University of Warwick Coventry CV4 7AL United Kingdom; b Diamond Light Source Harwell Campus Oxfordshire OX11 0DE United Kingdom; c ISIS Neutron and Muon Facility Rutherford Appleton Laboratory Didcot OX11 0QX United Kingdom; The University of Western Australia, Australia

**Keywords:** pressure transmitting mediums, molecular dynamics, hydrostaticity, high-pressure crystallography

## Abstract

We employ a combined computational and experimental approach to systematically assess the hydrostatic properties of methanol–ethanol (MeOH–EtOH) mixtures of varying compositions, with the aim of evaluating their suitability as pressure-transmitting mediums (PTMs). PTMs are essential for enabling the characterization of materials properties at high pressure, perhaps most prominently in the context of diffraction measurements, to provide uniform compression and avoid strain on the sample. Molecular dynamics (MD) simulations indicate that the hydrostatic limit and several structural and dynamic properties of the widely used 4:1 MeOH–EtOH volume ratio do not exhibit any significant deviations from the monotonic trends observed as a function of MeOH content within the mixture. These findings are in agreement with X-ray pair distribution function measurements, which show no peculiar structural behaviour for the 4:1 composition. Experimental measurements of the hydrostatic limit confirm this result and demonstrate that, as previously reported, the role of ethanol is primarily to delay MeOH crystallization. However, we find that the same role can be fulfilled by other small molecules, such as propan-2-ol. Additional simulations of several MeOH–*X* binary mixtures suggest that these results might hold for a variety of similar mixtures. Thus, our findings indicate that the 4:1 ratio is neither peculiar nor optimal in terms of PTM performance; instead, its popularity seems to be mostly due to the influence of previous literature. Indeed, we find that the 9:1 MeOH–EtOH mixture is characterized by a hydrostatic limit which is superior (by nearly 1 GPa) to that observed for the 4:1 ratio. These findings offer a promising alternative PTM composition which is readily available, and pave the way towards future work aimed at the rational design of novel PTMs.

## Abstract 2

In this combined computational and experimental study, we demonstrate that, despite its widespread use, a methanol–ethanol ratio of 4:1 is not optimal for use as a pressure-transmitting medium. Consistent with previous reports, we confirm that ethanol primarily serves to delay crystallization. However, our results also demonstrate that different components and ratios can offer modestly higher hydrostatic limits.

## 1. Introduction

Understanding the behaviour of materials under pressure can reveal novel and exotic states of matter, including high-pressure superconductors, high-energy-density materials and new porous materials suitable for high-pressure gas storage (Xu *et al.*, 2022 ▸; Sun *et al.*, 2021 ▸; Li *et al.*, 2018 ▸; Erdős *et al.*, 2021 ▸). Structural changes at high pressure also have significant implications for drug processing (Gasol-Cardona *et al.*, 2025 ▸) and can enhance our understanding of geophysical and astronomical processes (Maynard-Casely, 2017 ▸). Supported by recent advances in laboratory-based high-pressure equipment and the development of dedicated experimental beamlines, high-pressure research is one of the most rapidly expanding areas of crystallography (Motaln *et al.*, 2025 ▸; Wilson *et al.*, 2022 ▸).

Experimental methods using opposed-anvil cells to study materials at high pressure apply pressure in only one direction. A full description of a high-pressure experiment can be found in the work of Katrusiak (2008 ▸). Although pressure is fundamentally a scalar quantity by definition, it can be associated with forces acting at specific positions and in specific directions (Lewin, 2015 ▸; Richards, 2001 ▸; Oertel, 2004 ▸). Within the formalism of classical mechanics, the ‘pressure tensor’ is defined as the Cauchy stress tensor taken with the opposite sign, *i.e.P*_*ij*_ = −σ_sim_ (Shi *et al.*, 2023 ▸). More precisely, pressure is described using a tensor where each component *P*_*ij*_ represents a force acting in direction *j* on a surface normal to direction *i* (Richards, 2001 ▸; Oertel, 2004 ▸). Alternatively, the forces contributing to the pressure can act in multiple directions and locations. For example, thermodynamic pressure accounts for all normal forces acting on the surface of a thermodynamic system (Lewin, 2015 ▸). Thus, if the goal is to ensure a given value of thermodynamic pressure, studying a material under high pressure requires the sample to be surrounded by a pressure-transmitting medium (PTM). The PTM ensures that all normal pressure components *P*_*ii*_ are equal, mitigating against the uniaxial geometry of the cell and providing uniform pressure from the diamond anvils as it is progressively increased during the experiment (Katrusiak, 2019 ▸). When a PTM allows for the *P*_*ii*_ components to remain equal, it is said to be hydrostatic.

The choice of PTM presents several experimental challenges. It must not interact with the sample (reaction or solvation), contamination of the diffraction signal should be minimal, it must not enter any pores in the sample, and it must deliver isotropic (hydrostatic) compression over the entire pressure range of interest. Commonly used PTMs include liquids and gases, but the difficulties and cost associated with using specialist gas-loading equipment often leave liquid PTMs the preferred choice (Motaln *et al.*, 2025 ▸). The upper pressure limit at which a PTM is able to impart isotropic compression is referred to as its hydrostatic limit, and is often associated with solidification. Manifesting as either crystallization or vitrification of the liquid, solidification remains the dominant limitation in currently available liquid PTMs. Despite the importance of PTMs in high-pressure experiments, surprisingly little work has been devoted to the discovery of new PTMs, largely due to the technical difficulty and time-consuming nature of the experiments. Improved understanding at the molecular level of the behaviour of PTMs at their hydrostatic limit could significantly simplify this process.

The most common choice of PTM is a 4:1 volume ratio of methanol (MeOH) and ethanol (EtOH) (Piermarini, 2001 ▸). With a hydrostatic limit of 10.5 GPa, it remains the state-of-the-art PTM for non-porous materials (Klotz *et al.*, 2009 ▸). The origins of the specific ratio, however, are not clear from the literature. MeOH was first reported to undergo a ‘sluggish’ crystallization at a pressure of 2.94 GPa, which could occasionally be bypassed, while EtOH was first reported to crystallize at 1.96 GPa – both at 25°C (Bridgman, 1942 ▸). These values have been re-evaluated several times since. For EtOH, crystallization pressures fall in a narrow range, between 1.78 GPa (Mammone *et al.*, 1980 ▸) and 1.9 GPa (Shimizu *et al.*, 1990 ▸). For MeOH, a greater variation has been reported. It was next reported to crystallize at a slightly higher pressure of at least 3.8 GPa, through linear extrapolation of shifts in the absorption band of nickel dimethylglyoxime (Davies, 1968 ▸). This was again revised to 3.58 GPa (Piermarini *et al.*, 1973 ▸) and then more recently to 3.5 GPa (Mammone *et al.*, 1980 ▸). Through the various reported crystallization pressures for MeOH, Piermarini *et al.* (1973 ▸) realized the ease with which the crystallization point of MeOH could be suppressed. Under rapid compression they reported that the crystallization of MeOH can be suppressed entirely, observing what was believed to be a glass transition at 8.6 GPa. Piermarini *et al.* were the first to suggest the use of MeOH–EtOH mixtures; they aimed to inhibit crystallization of pure MeOH by introducing a hydrogen-bond competition. Their choice of the 4:1 ratio was found to extend the glass transition of the mixture to 10.4 GPa.

Later contributions cast into doubt the reported glass transition of MeOH. The structure in this pressure range was shown through viscosity measurements to be inconsistent with a glass (Cook *et al.*, 1993 ▸). Their measurements predicted that the glass would not be observed until 11.0 GPa, while reproducing the glass transition in the 4:1 mixture at 10.5 GPa. These findings were further supported by Brugmans & Vos (1995 ▸) who, through optical microscopy and diffusion measurements, found that crystals nucleate most effectively between 5 and 10.5 GPa, with a maximum at 7.0 GPa. They observed the formation of a glass only above 10.5 GPa, and only provided the nucleation range was passed quickly enough, in good agreement with the viscosity measurements. In turn, this argument was used to conclude that the previously reported glass transition at 8.6 GPa was probably a delayed crystallization.

The glass transition of 4:1 MeOH–EtOH has since been measured in several additional reports in the range 9.8–10.8 GPa (Angel *et al.*, 2007 ▸; Piermarini *et al.*, 1973 ▸; Klotz *et al.*, 2009 ▸; Eggert *et al.*, 1992 ▸). The role of water in the mixture has also been previously investigated, following a claim that a 16:3:1 ratio of MeOH–EtOH–H_2_O increases the hydrostatic limit to 14.5 GPa (Jayaraman, 1983 ▸; Fujishiro, 1982 ▸). This has since been disproven and a 16:3:1 mixture was measured to have a hydrostatic limit, within error, of 10.5 GPa (Klotz *et al.*, 2009 ▸; Angel *et al.*, 2007 ▸). The role of deuteration was also found to have no impact on the hydrostatic limit of either the 4:1 or 16:3:1 ratios (Klotz *et al.*, 2009 ▸). Rather, the glass transition of a mixture is largely dictated by the glass transition of its most abundant component. Thus, as previously reported, the role of EtOH in the PTM mixture is merely to hinder the crystallization of MeOH, as originally intended, without providing any improvements to the vitrification pressure (Cook *et al.*, 1993 ▸). Despite this, the benefits of this ratio are still widely reported within the literature. It has been suggested that slight inaccuracies in solution preparation may cause minor deviations in the actual mixing ratio, which could in turn reduce the glass-transition pressure and help explain the range of hydrostatic limits reported in the literature, but this possibility has not been thoroughly investigated (Takemura, 2021 ▸; Fujishiro, 1982 ▸). The importance of the ratio itself remains unclear, as it is not currently understood whether it corresponds to a peculiar set of structural or dynamic properties of the mixture, or if its usage is purely down to historical precedent.

In this work, we investigate the hydrostatic properties of MeOH–EtOH mixtures in various compositions by means of a joint computational and experimental approach. To guide experimental investigations, molecular dynamics (MD) simulations were first performed on MeOH–EtOH mixtures to explore whether any unique structural behaviour or hydrostatic limit is associated with the 4:1 volume ratio. Structural results stemming from these simulations were validated using experimental X-ray pair distribution function (XPDF) measurements and the hydrostatic limits of selected compositions were subsequently determined experimentally. The relevance of EtOH as a component of the mixture was assessed by replacing it with propan-2-ol (*i*-PrOH). Further MD simulations across a broader range of ethanol substitutes show that these conclusions might also be applicable to a variety of similar MeOH-based mixtures.

## 2. Methods

### 2.1. Molecular simulations

MeOH–EtOH mixtures in volume ratios of 1:9, 1:4, 3:7, 2:3, 1:1, 3:2, 7:3, 4:1 and 9:1, as well as pure MeOH and pure EtOH, were investigated by means of classical MD simulations. Each system was first studied at ambient temperature (300 K) and pressure (1 atm), aiming to see whether any particular composition displays exceptional behaviour under these conditions. Subsequentially, the systems were then subjected to progressive isothermal uniaxial compression up to 12 GPa, with the aim of mimicking the experimental setup described below. Starting from ambient pressure, the normal pressure component in the arbitrarily chosen *z* direction, *P*_*zz*_, was increased abruptly by 0.2 GPa every 2 ns. Several force fields were considered in the study to ensure the robustness of the results. Specifically, four different force fields, namely CGenFF, OPLS-AA, TraPPE and the force field proposed by Guevara-Carrion *et al.* (2008 ▸), were considered in the study under ambient conditions (Vanommeslaedhe *et al.*, 2010 ▸; Jorgensen *et al.*, 1996 ▸; Chen *et al.*, 2001 ▸; Guevara-Carrion *et al.*, 2008 ▸; Schnabel *et al.*, 2007 ▸; Schnabel *et al.*, 2005 ▸). However, only CGenFF and OPLS-AA were used for the study under uniaxial compression, since the TraPPE and Guevara-Carrion *et al.* (2008 ▸) force fields include intra­molecular constraints that may prevent meaningful simulations at high pressure. The TraPPE and Guevara-Carrion *et al.* (2008 ▸) force fields are still valuable in the investigation under ambient conditions, though, as these are more specifically parametrized towards EtOH and MeOH than are OPLS-AA and CGenFF.

Configurations sampled from the last nanosecond of the trajectories at each given pressure were used to calculate the properties of interest. Under ambient conditions, we have computed continuous hydrogen-bond lifetime, angle distribution functions, coordination numbers, pair distribution functions (PDFs), densities and self-diffusion coefficients. Under uniaxial compression we have computed PDFs, densities and self-diffusion coefficients. Average values of the normal pressure components in the *x* and *y* directions, *P*_*xx*_ and *P*_*yy*_, respectively, were additionally estimated to evaluate whether they are equivalent to the enforced value of *P*_*zz*_ and thus to determine whether the system is hydrostatic. This is quantified by the parameter σ_sim_:
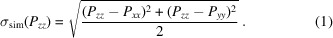
We chose this method to assess hydrostaticity as it assesses it fundamentally on its definition (*i.e.* its ability to transmit pressure *per se*) without any assumption on phase transitions. Specifically, we are enforcing a given component *P*_*ii*_ of the pressure tensor and evaluating the ability of the system to respond to such pressure by reaching a new corresponding equilibrium state, where other *P*_*jj*_ components are equal to the enforced *P*_*ii*_. Naturally, however, loss of hydrostaticity is linked to solidification: liquids and gases, as stated by Pascal’s law, can redistribute pressure thanks to molecular motion, whereas solids cannot, due to the absence of significant translational motion. Thus, the method can be regarded as an indirect evaluation of the pressure at which solidification occurs. Three different values of σ_sim_ were used as the threshold for determining when hydrostaticity is lost upon compression. Further details of the MD simulations and of the estimation of the different properties can be found in the supporting information.

Finally, to study the relevance of EtOH within the mixtures, additional MeOH-based binary mixtures were also studied via MD. The volume ratio for these mixtures was fixed to that yielding the highest hydrostatic limit among those investigated for the MeOH–EtOH mixtures. Compounds of different molecule sizes, chemistry and hydrogen-bond propensity were chosen. These were propan-2-ol, acetone, propanal, acetic acid, formic acid, acetonitrile, ethylene glycol, 1,2-di­meth­oxy­ethane, benzene, pyridine, dimethyl sulfoxide, octanol, cyclooctanol, tetrahydrofuran and *N*-methyl-2-pyrrolidone. The same MD simulation protocol as deployed for studying MeOH–EtOH at high pressure was used, but only the values of *P*_*xx*_ and *P*_*yy*_, and ultimately σ_sim_, were calculated for these mixtures to assess their hydrostatic limit.

### 2.2. Experimental determination of hydrostatic limits

Due to concerns raised in the literature about the role of water in MeOH–EtOH mixtures, efforts were made to ensure the highest possible purities of all starting solvents while retaining practical relevance. In each case, new solvent stocks were used. Absolute EtOH (VWR), MeOH (≥99.8%, Fisher Chemical) and propan-2-ol (≥99.8%, Sigma Aldrich) were used as supplied. MeOH is highly susceptible to water uptake, and therefore the water content was continuously mapped by hydrometry (Brannan), remaining within error of 0% throughout this study. The experimentally studied PTMs were MeOH, EtOH and MeOH–EtOH mixtures in volume ratios of 1:1, 4:1 and 9:1, as well as a 9:1 MeOH–*i*-PrOH mixture. To improve resolution and repeatability in the determination of the hydrostatic limit of the promising 9:1 MeOH–EtOH mixture, an additional loading of this mixture was performed. All mixtures were prepared using a micropipette.

High-pressure measurements were completed within Almax easyLab Diacell Bragg-Mini diamond anvil cells (DACs) with an opening angle of 85°. The cell design is based on the standard Merrill–Bassett design (Merrill & Bassett, 1974 ▸). The DACs consisted of conical tungsten carbide backing plates (Moggach *et al.*, 2008 ▸) and Boehler–Almax type IIas design (Boehler & De Hantsetters, 2004 ▸) conical diamond anvils featuring 500 µm diameter culets. Tungsten gaskets of 200 µm diameter were indented to 60 µm in all experiments. It was found that a 60 µm indent provided better pressure control and greater resistance to deformation of the gasket than a shallower indent. The sample chamber was drilled with a BETSA MH20M electric discharge machine with a 250 µm diameter electrode.

**Figure 1:**
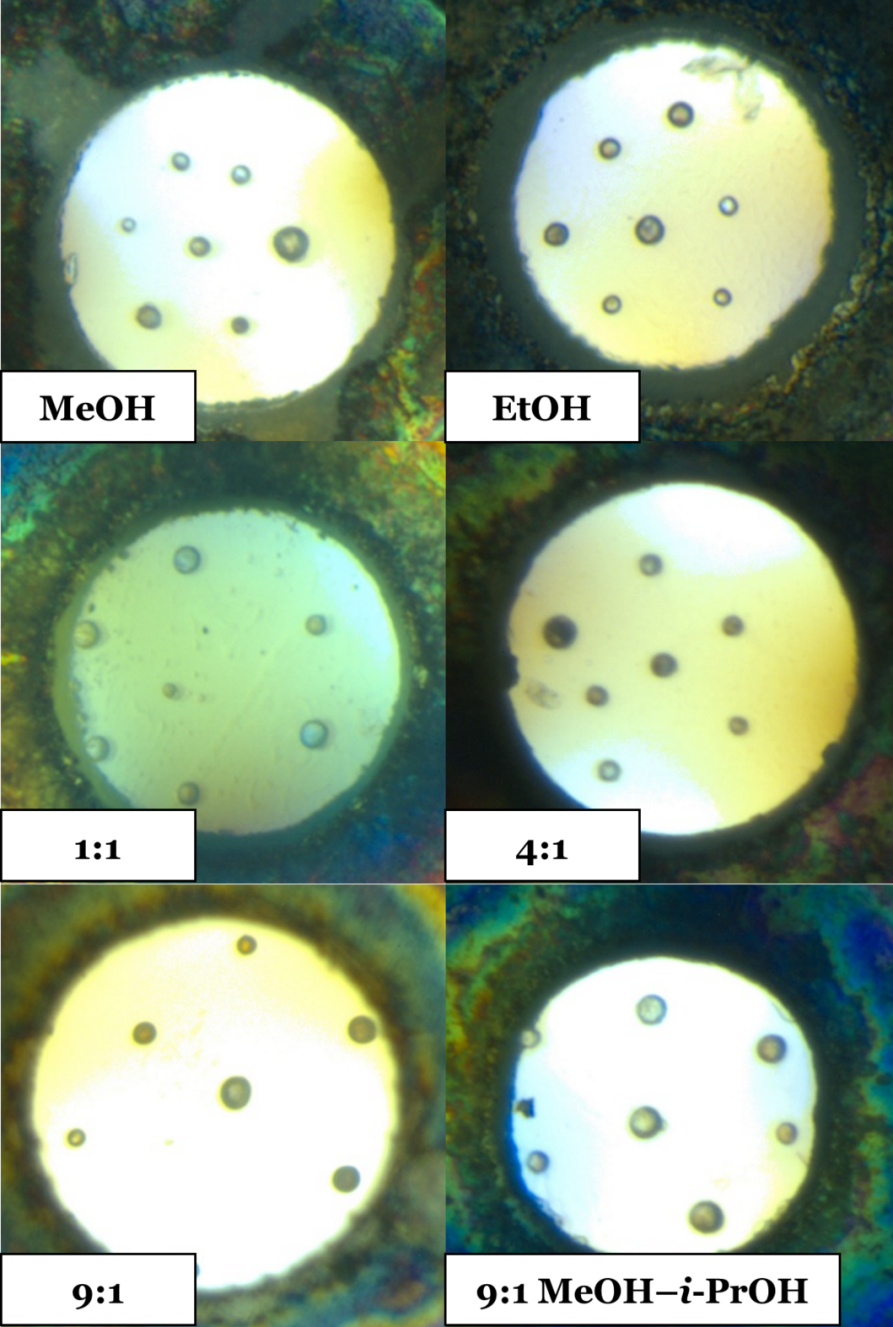
DAC sample chambers. (Left to right, top to bottom) MeOH, EtOH, 1:1 MeOH–EtOH, 4:1 MeOH–EtOH, 9:1 MeOH–EtOH and 9:1 MeOH–*i*-PrOH.

The measurements of the hydrostatic limit presented here closely follow the method of Klotz *et al.* (2009 ▸), whereby the onset of non-hydrostatic conditions is mapped by the increase in the standard deviation σ of pressure values measured from a distribution of rubies across the sample chamber. In each experiment the sample chamber was loaded with seven polycrystalline ruby spheres [(Al_2_O_3_):Cr^3+^, 3–50 µm, supplied by BETSA] spaced across the upper culet, as shown in Fig. 1 ▸ (an image of the sample chamber for the second loading of 9:1 MeOH–EtOH can be found in the supporting information). The ruby spheres supplied had undergone previous heat treatment to provide sharper *R*_1_ and *R*_2_ lines for more accurate pressure calibration. Measurement of *R*_0_, *i.e.* the ambient-pressure fluorescent wavelength of each ruby, and all subsequent wavelength measurements were performed with an Almax easyLab Optiprexx photoluminescence device, a stand-alone spectrometer featuring a 532 nm 50 mW laser. The cumulative error in the pressure measurements is equivalent to ±0.045 GPa. The *R*_0_ values varied between 694.289 and 694.320 nm. Individual *R*_0_ values were used for each ruby to calculate the pressure using the ruby fluorescence method (Chervin *et al.*, 2001 ▸).

The PTM being investigated was added via syringe and, once the absence of bubbles in the sample chamber was confirmed, the cell was rapidly closed. Uniaxial pressure was applied in the smallest achievable increments. A minimum of 15 min was allowed to elapse after each pressure increase before measurements were made. Measurements were then repeated at regular intervals until cessation of the pressure drift, at which point another pressure increase was made. At each pressure point the fluorescent wavelength of each of the rubies was measured in turn, the temperature recorded and the pressure calculated. The average pressure was then mapped against the standard deviation σ in the pressures, calculated as previously described (Klotz *et al.*, 2009 ▸). After initial optical centring, the signal was maximized for the highest quality fit (further details of the experimental method are available in the supporting information).

When the highest pressure of any ruby within the cell was within an error of 20 GPa, the highest safe loading pressure for the DACs in use, the cell was decompressed. Decompression points were used to demonstrate a recovery of hydrostatic conditions within the cell, such that any loss of hydrostaticity could be attributed solely to the PTM and not to irreversible damage to the gasket or cell. Motaln *et al.* (2025 ▸) observed changes in the ruby fluorescence signal which were attributed to the prolonged exposure of rubies to non-hydrostatic pressure, and therefore in our study ruby spheres were not reused between experiments.

### 2.3. Experimental PDF analysis

XPDF measurements were completed on beamline I15-1 at Diamond Light Source at a wavelength of 0.161669 Å at room temperature. Liquid samples of MeOH, EtOH and MeOH–EtOH mixtures with ratios 4:1, 3:2, 1:1, 2:3 and 1:4 were prepared in 2 mm borosilicate glass capillaries with an inner diameter of 1.56 mm. Data were processed using *GudrunX* (Soper, 2011 ▸) and consistent parameters were used for all samples. A top hat convolution was applied with a width of 3 Å^−1^ and a minimum radius for the Fourier transform of 0.8 Å. Using this correction, and due to the weakly scattering nature of the samples, the range of *Q* was cut to a *Q*_max_ of 18 Å to remove data which suffered a significantly poorer signal-to-noise ratio and did not benefit the overall PDF.

## 3. Results and discussion

### 3.1. MeOH–EtOH mixtures: the importance of composition

**Figure 2:**
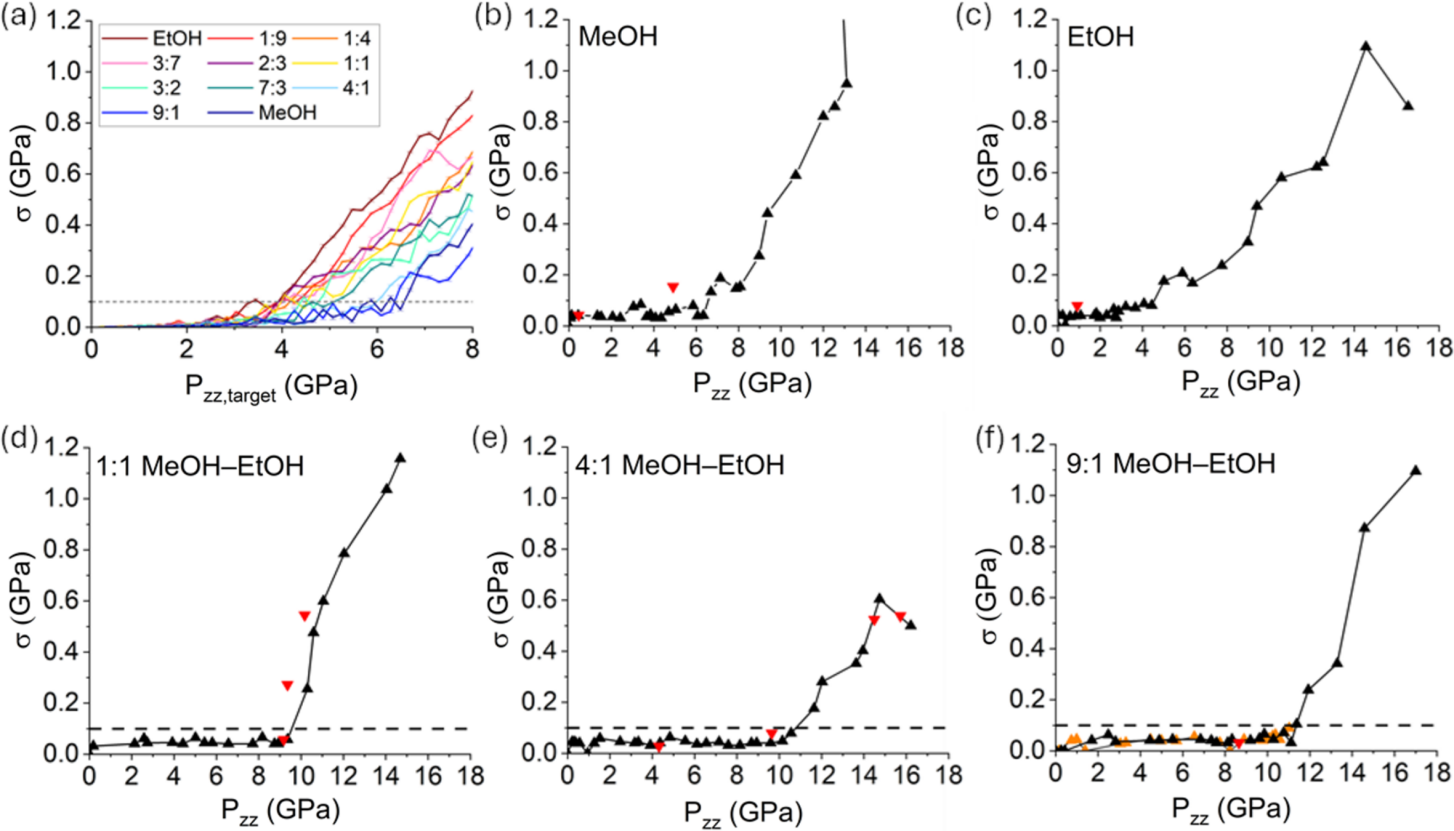
Hydrostatic limits of different MeOH–EtOH mixtures. Values of the standard deviation σ obtained at different pressures in (*a*) the simulated pure MeOH, pure EtOH and their mixtures, and experimentally for (*b*) MeOH, (*c*) EtOH, (*d*) 1:1 MeOH–EtOH, (*e*) 4:1 MeOH–EtOH and (*f*) 9:1 MeOH–EtOH. In the experimental plots, black upward triangles represent compression points and red downward triangles represent decompression points. In panel (*f*) orange upward triangles represent the second loading. All experimental plots are presented on the same axis scale for direct comparison. Dashed lines are used in panels (*a*), (*d*), (*e*) and (*f*) to indicate the consistent value at which σ is observed to increase sharply.

Fig. 2 ▸(*a*) presents the values of σ_sim_ at each imposed *P*_*zz*_ as obtained from the MD simulations for all investigated MeOH–EtOH compositions. The corresponding hydrostatic limits, obtained considering the *P*_*zz*_ value at which a value σ_sim_ = 0.1 is first reached, are presented in Table 1 ▸. Only the results obtained using CGenFF are shown. The same conclusions hold when using OPLS-AA to model the interactions (see the supporting information for an equivalent plot obtained using the OPLS-AA force field and hydrostatic limits with a wider range of σ values for both force fields). In Table 1 ▸, the simulations point towards the 9:1 and 4:1 MeOH–EtOH mixtures having a higher hydrostatic limit than pure MeOH. However, as can be seen in Fig. 2 ▸(*a*), the evolution of σ_sim_ with *P*_*zz*_ for MeOH near σ_sim_ = 0.1 oscillates. This suggests that pure MeOH might have a higher hydrostatic limit than both the previously mentioned mixtures, despite first reaching the σ_sim_ = 0.1 threshold at a lower *P*_*zz*_ value. Thus, these results support the trend that the hydrostatic limit increases with MeOH content. No unique features relative to any particular composition are visible. Note that these systems will never crystallize within the timescale explored by unbiased MD simulations and thus we can only computationally probe the vitrification of the mixtures.

**Figure 3:**
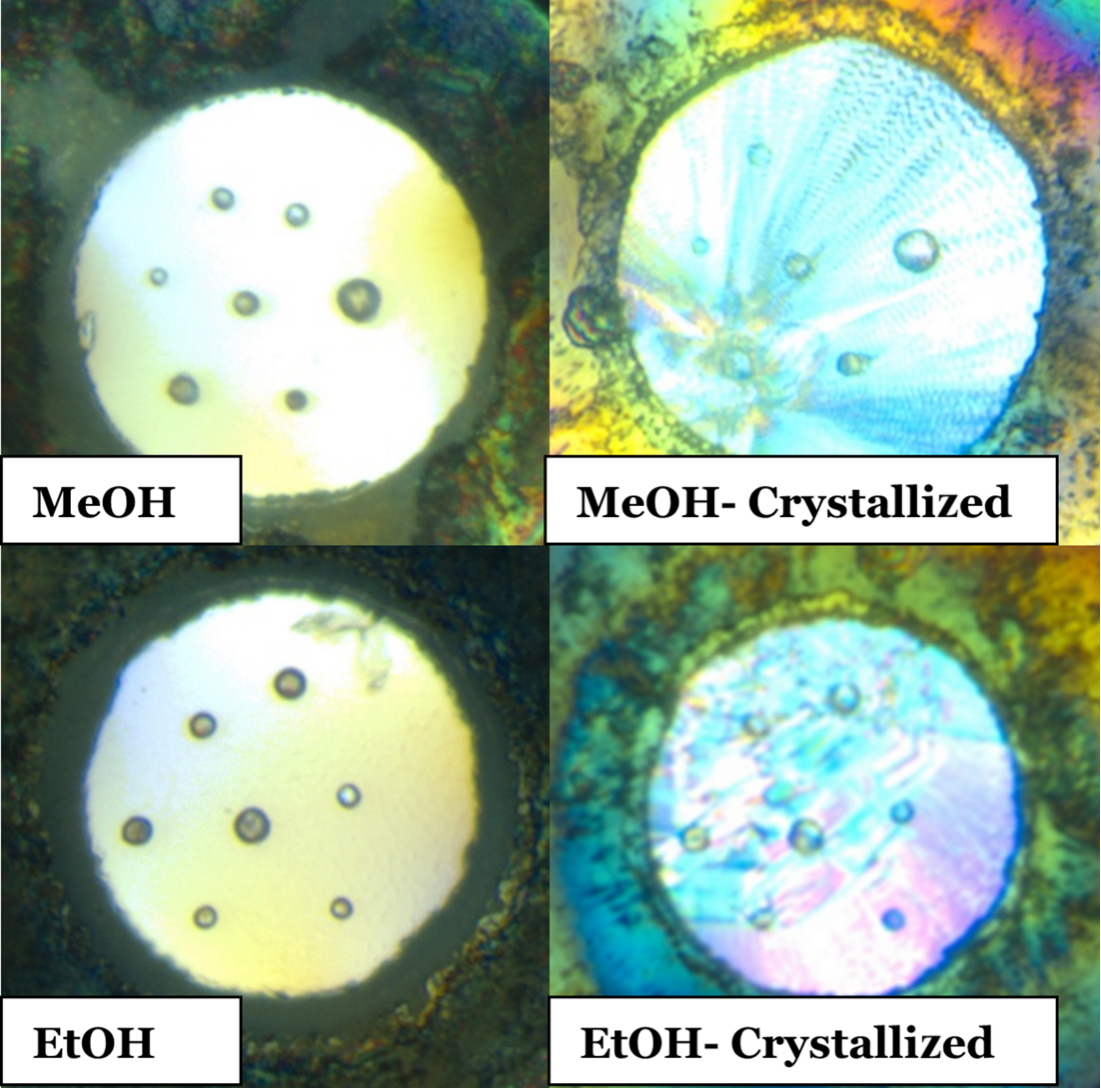
Single-component PTMs which crystallized on compression. (Top left) Pure MeOH at ambient pressure and (top right) frozen at 6.70 GPa. (Bottom left) Pure EtOH at ambient pressure and (bottom right) frozen at 2.71 GPa.

To validate these results, we have experimentally investigated the hydrostatic limits of pure MeOH, pure EtOH and a number of their mixtures. Figs. 2 ▸(*b*)–2(*f*) display the evolution of σ with uniaxial compression and again Table 1 ▸ summarizes their hydrostatic limits. Figure panels are presented on the same axes for direct comparison (a graph displaying all measured pressure points for MeOH is available in the supporting information). Both loadings of 9:1 MeOH–EtOH are presented on the same graph but in different colours. The hydrostatic limit reported in each case corresponds to either the crystallization pressure or the pressure that occurs immediately before a steep increase in σ is observed, using the same criterion as described above. In the latter case, the loss of hydrostaticity is assumed to be related to vitrification. Pure MeOH was observed to crystallize unambiguously by optical microscopy at 6.70 GPa, very close to the pressure where the maximum nucleation rate is observed, as reported by Brugmans & Vos (1995 ▸). Fig. 3 ▸ shows the sample chamber before and after crystallization. Crystallization of the methanol is obvious from the striations across the sample and the increased opacity of the sample chamber. This indicates a super-press of MeOH (Piermarini *et al.*, 1973 ▸; Reeves *et al.*, 1964 ▸; Gelles, 1968 ▸), as is widely reported in the literature, given its slow compression crystallization pressure is 3.5 GPa (Mammone *et al.*, 1980 ▸; Piermarini *et al.*, 1973 ▸; Davies, 1968 ▸; Bridgman, 1942 ▸). A small deformation of the gasket occurred at 13.10 GPa, and here an increase in volume of the sample chamber resulted in a small drop in average pressure to 12.91 GPa. On decompression from 15.62 to 4.90 GPa, the MeOH remained frozen, and it was only on returning to 0.46 GPa that it was first observed to have melted. At this point, σ returned to its previous level, indicating that any loss in hydrostaticity could be solely attributed to the PTM and not to the gasket deformation.

Pure EtOH was observed to crystallize at 2.71 GPa, analogously to MeOH, again by optical microscopy (Fig. 3 ▸). There have been fewer studies of EtOH under compression (Bridgman, 1942 ▸; Mammone *et al.*, 1980 ▸; Shimizu *et al.*, 1990 ▸), but it probably follows the profile of crystallization of a super-pressed liquid (Piermarini *et al.*, 1973 ▸; Reeves *et al.*, 1964 ▸; Gelles, 1968 ▸), very similar to MeOH, as the crystallization pressure of EtOH under slow compression is reported to be 1.96 GPa. A further discontinuity at this pressure reflects a decrease of 0.15 GPa on crystallization of the sample. For EtOH, the increase in the standard deviation was initially more subtle before more rapidly increasing above 4.43 GPa. At the highest pressure point, 16.54 GPa, the σ value reduced. This is similar to the behaviour seen by Klotz *et al.* (2009 ▸) and Motaln *et al.* (2025 ▸) and is attributed here to instabilities within the cell rather than genuine features. On decompression to 0.91 GPa, hydrostatic conditions were recovered.

All MeOH–EtOH mixtures investigated experimentally displayed very similar behaviour under compression, with the steep increase in σ being associated with vitrification of the sample. All ratios display a hydrostatic limit in a relatively narrow range (Table 1 ▸). In the second loading of the 9:1 mixture, the cell failed upon compression beyond the hydrostatic limit observed in the first loading. Accordingly, this loading serves predominantly to improve the resolution in the determination of the hydrostatic limit. Notably, the hydrostatic limits computed by MD simulations are consistent with the experimental results. Due to the fact that the compression rates we are forced to use in our simulations are several orders of magnitude higher than those utilized in the experimental protocols, the computational estimates of the hydrostatic limit for any system are bound to be underestimated. However, we argue that this might very well be a systematic error, on the basis of similar considerations on the vitrification of molecular glasses upon supercooling (as opposed to compression) (Barnard & Sosso, 2023 ▸). In the results presented here, we see an average offset of 4.91 GPa between the experimental and simulated data, and this has been used to scale all simulated vitrification pressures in Table 1 ▸. The 1:1 ratio has an experimental hydrostatic limit of 9.36 GPa and the 4:1 ratio has an experimental hydrostatic limit of 10.56 GPa, the latter in very good agreement with the literature. The 9:1 ratio displays the best hydrostatic behaviour, showing a 0.8 GPa improvement in hydrostatic limit over the 4:1 ratio. Remarkably, it appears that by using a 9:1 ratio instead of a 4:1 ratio almost an entire additional 1 GPa of hydrostatic conditions can be gained without compromising the ability of the mixture to stall crystallization. Further stability tests against the crystallization of the 9:1 mixture can be found in the supporting information. While we note that, on the basis of the trend observed in this work, mixtures even richer in MeOH may offer a further improvement in the hydrostatic limit, we stress the importance of keeping the EtOH content high enough to ensure that the mixture remains liquid at high pressures.

#### 3.1.1. Structural analysis

**Figure 4:**
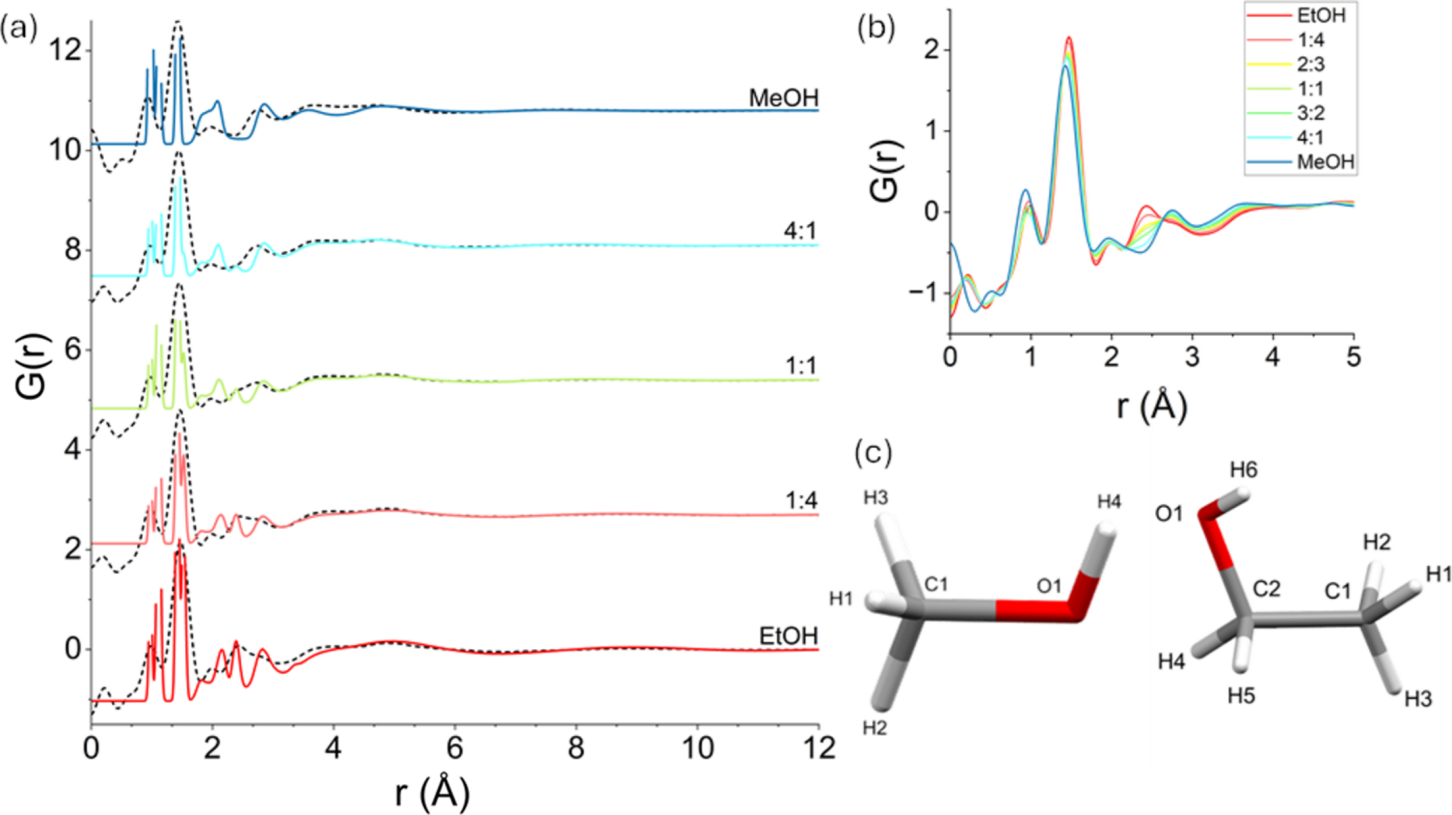
Structural properties of MeOH–EtOH mixtures at ambient pressure. The PDFs for different MeOH–EtOH mixtures under ambient conditions obtained from (*a*) simulations and experiments and (*b*) experiments only, overlapped for comparison. Panel (*a*) shows the change in the simulated *G*(*r*), from red to blue curves, as the MeOH fraction increases. Experimental *G*(*r*) are represented by black dashed lines. The different systems are offset vertically for better visualization and the heights of the peaks in the simulated PDFs were truncated for clarity whenever they extended beyond the experimental PDF for the given system (*i.e.* peaks with *r* < 2 Å). Panel (*c*) illustrates the naming convention used for each atom in the MeOH and EtOH molecules. Molecular diagrams drawn using *Mercury* (Groom *et al.*, 2016 ▸).

Analysis of both simulated and experimental PDFs reveals a lack of structural anomalies for any of the investigated mixtures. Fig. 4 ▸(*a*) shows the experimental PDFs overlaid with the simulated PDFs for EtOH, MeOH, and the 1:4, 1:1 and 4:1 MeOH–EtOH mixtures at ambient pressure and temperature. All data are normalized using the *G*(*r*) convention (Keen, 2001 ▸). Curves for different compositions have been vertically offset for clarity. Similar results obtained via the OPLS-AA force field can be found in the supporting information. At physically meaningful distances (≥0.9 Å), all experimental PDFs are reasonably well reproduced, validating the quality of the force field. The experimental PDFs suffer peak broadening due to a limited *Q*_max_ range, whereas the computational PDFs do not. Fig. 4 ▸(*b*) overlays all the experimental PDFs, including those for the 3:2 and 2:3 MeOH–EtOH mixtures. The PDFs exhibit mostly a monotonic trend, with each mixture’s profile closely resembling that of its dominant component. As the MeOH fraction increases, the PDFs progressively shift from resembling EtOH to resembling MeOH.

The computational PDFs provide insights into the contributions of atom pairs to the individual peaks of the PDFs shown in Figs. 4 ▸(*a*) and 4 ▸(*b*). Hydrogen atoms are generally not observed in XPDF measurements due to the small scattering cross section of hydrogen to X-rays. Despite this, some of the peaks observed experimentally can only be assigned to pairs of atoms involving hydrogen. Tests performed to ensure that these peaks cannot be attributed to artefacts of the Fourier transform using different *Q*_max_ values did not cause these peaks to shift until a significant degradation of the entire PDF occurred. These peaks are the result of the absence of strongly scattering atoms in the mixtures, the reduced intermolecular pair distance contributions of the liquids and the high concentration of H in the samples.

The peak at ∼0.9 Å corresponds to O—H and C—H intramolecular bonds (lengths of approximately 0.98 and 1.12 Å, respectively) in both MeOH and EtOH. Any deviation from monotonic behaviour with respect to this peak as the composition changes in the experimental PDF can be attributed to the sensitivity of the technique. The most intense peak in all the PDFs can be found at approximately ∼1.45 Å. According to the labelling scheme illustrated in Fig. 4 ▸(*c*), for EtOH-containing systems this peak has its origins in the C2—O1 (1.42 Å) and C1—C2 (1.53 Å) intramolecular bonds, while in MeOH-containing systems only the C1—O1 (1.43 Å) intramolecular bond contributes to it. The peak reduces in intensity and shifts from right to left as the fraction of MeOH increases. The peak at ∼1.95 Å is similar for the two compounds and stems primarily from intermolecular hydrogen bonds. In EtOH this is the H6⋯O1 hydrogen bond and in MeOH it is the H4⋯O1 hydrogen bond, where the O1 atom in both cases can be from either an MeOH or an EtOH molecule in the mixtures. These have average bond lengths of approximately 1.89 Å. In addition, longer H⋯C, H⋯H and H⋯O pair distances also contribute, albeit to a lesser extent, to this peak in both pure compounds. Given the importance of hydrogen bonds for these systems, the fact that this peak reveals no structural abnormality for 4:1 MeOH–EtOH, but rather a monotonic trend, suggests a lack of peculiar features for the 4:1 ratio. Note that the peak at 2.46 Å is present in EtOH-contaning systems but completely absent from pure MeOH. This corresponds primarily to the intramolecular C1⋯O1 pair distance in EtOH, which is not present in MeOH. The peaks beyond 2.7 Å are weaker and mostly carry a contribution from intermolecular pair distances. The same conclusions concerning the atomistic origins for the peaks in the experimental PDFs hold when the interactions are modelled using OPLS-AA (see the supporting information).

**Figure 5:**
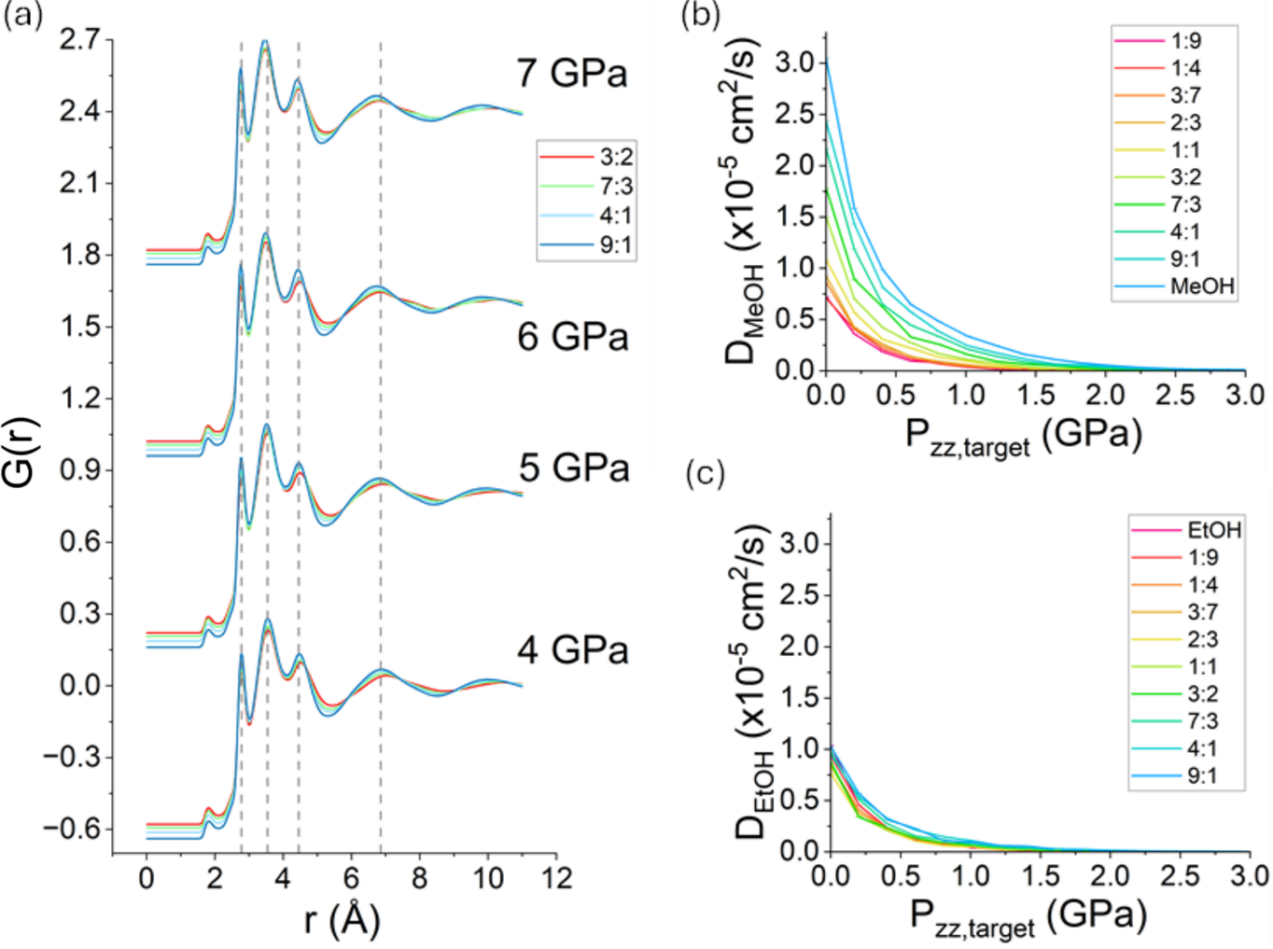
Structural properties of MeOH–EtOH mixtures upon compression. (*a*) Simulated PDFs for selected MeOH–EtOH mixture compositions under uniaxial compressions of 4, 5, 6 and 7 GPa, obtained via the CGenFF force field. Dashed vertical grey lines serve as a guide to the eye. (*b*) and (*c*) Self-diffusion coefficients *D* for (*b*) MeOH and (*c*) EtOH for different values of uniaxial compression. The plots shown in panels (*b*) and (*c*) feature the same *y*-axis range so as to highlight the difference between the self-diffusion coefficients of the two components.

Fig. 5 ▸(*a*) shows the simulated PDFs of the 3:2, 7:3, 4:1 and 9:1 MeOH–EtOH mixtures at 4, 5, 6 and 7 GPa with the interactions modelled using CGenFF. These values were chosen to encapsulate the range of hydrostatic limits predicted by the simulations for these systems. All intramolecular 1–2, 1–3 and 1–4 neighbours were excluded from the PDFs, thus ensuring that they describe primarily the intermolecular pair distances. This is particularly useful as specific features involved in phase transitions such as vitrification should originate from specific strutural features – such as the spatial arrangement of intermolecular (and not intra­molecular) pairs. A similar figure for OPLS-AA is presented in the supporting information and leads to the same conclusions as discussed herein. Assuming that the quality of the models in depicting the structure under ambient conditions is retained at higher pressures, the plots indicate that no anomalous behaviour emerges near the onset of hydrostaticity loss for any specific composition, including the 4:1 volume ratio.

#### 3.1.2. Origins of vitrification: self-diffusion coefficients

Figs. 5 ▸(*b*) and 5 ▸(*c*) illustrate the evolution of the self-diffusion coefficient *D* of MeOH and EtOH, respectively, as a function of the imposed *P*_*zz*_ at different compositions. While the results in Figs. 5 ▸(*b*) and 5 ▸(*c*) feature simulations using CGenFF, similar figures can be found in the supporting information for OPLS-AA, for which the same conclusions hold. The values of the self-diffusion coefficients are only presented up to values of *P*_*zz*_ where no further decrease in *D* with pressure was observed; deviations from this behaviour are assigned to the limitations of the methodology used to assess the diffusion (see details in the supporting information).

Given the link between the loss of hydrostaticity and the onset of vitrification, evaluating trends in dynamic properties can be valuable for assessing the degree of translational motion, which is significant in the liquid/gaseous state but not in the solid state. In the case of vitrification, *D* behaves like an order parameter. As the pressure increases, *D* is expected to drop by several orders of magnitude as the system becomes closer to the stability domain of the solid phase and draws away from that of the liquid phase, tending towards zero to signal a transition from a fluid to an arrested/glassy state. In Figs. 5 ▸(*b*) and 5 ▸(*c*) it is clear that the molecules of both MeOH and EtOH move progressively more slowly as the pressure increases. There is also a much greater variation in the diffusion rate of MeOH molecules than there is for EtOH mol­ecules. As the mixtures become richer in MeOH, the diffusion rate of MeOH increases. At low MeOH content, the diffusion of MeOH molecules approaches the values observed for EtOH. Here, a weaker trend is observed with respect to the MeOH content, and the diffusion of EtOH also seems to be faster as the mixture becomes richer in MeOH. This indicates, perhaps intuitively, that it is easier to diffuse through a sea of methanol than it is a sea of ethanol, as the smaller size of the former outweighs the stronger bonds it forms. The weaker dependency of *D*_EtOH_ on composition is such that some scatter in the trend is observed. Together, these results suggest that mixtures richer in MeOH are expected to remain hydrostatic at higher pressures than EtOH-rich mixtures, in agreement with the σ data. Again, no significant result for the 4:1 EtOH–MeOH mixture is observed relative to other compositions assessed.

### 3.2. MeOH-based binary mixtures: the irrelevance of EtOH

**Figure 6:**
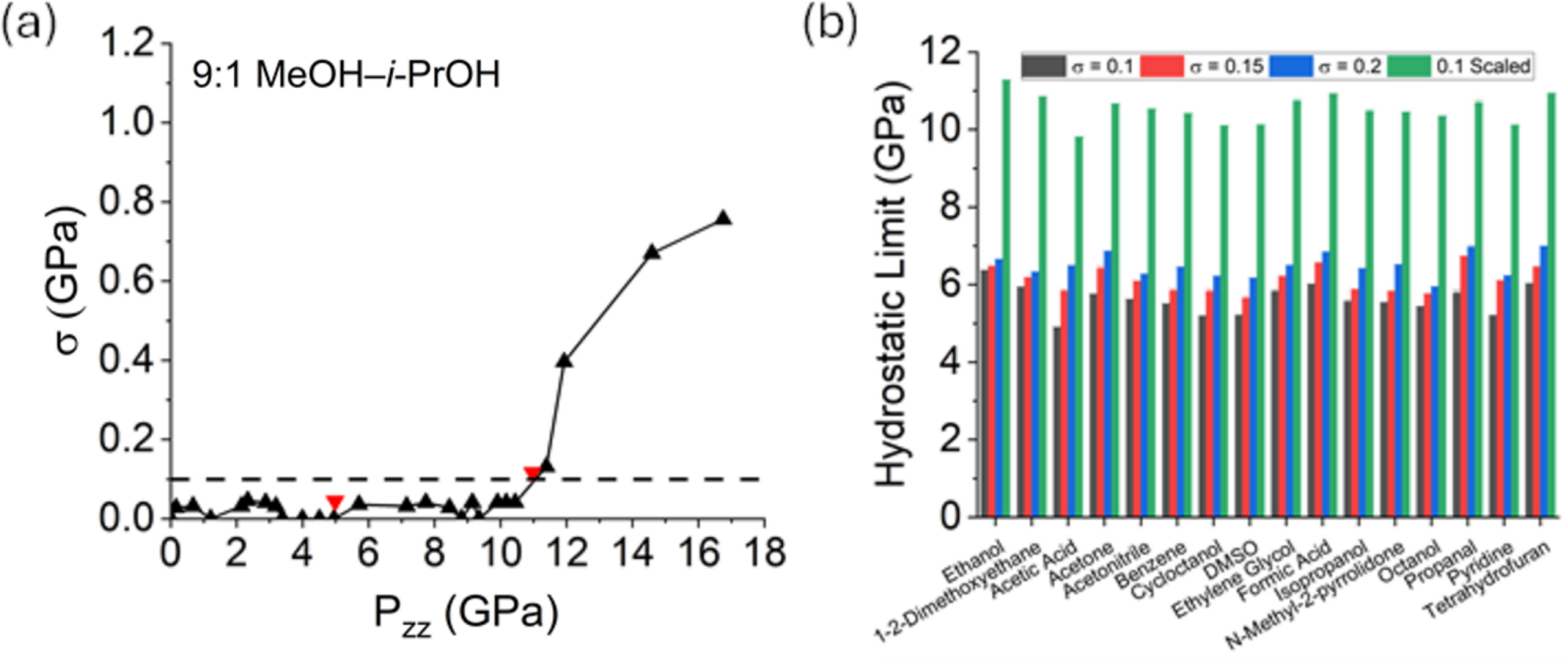
Hydrostatic limits for additional binary mixtures. (*a*) Evolution of the standard deviation with uniaxial compression for the 9:1 MeOH–*i*-PrOH mixture observed experimentally, presented on the same axes as Fig. 2 for direct comparison. Black upward triangles represent compression points, red downward triangles represent decompression points and the dashed line indicates the consistent value of σ at which it is observed to increase sharply. (*b*) Hydrostatic limits observed for all the 9:1 volume ratio MeOH-based mixtures that were simulated (the component in the smaller fraction is indicated on the *x* axis). The data were determined using as criterion σ_sim_ = 0.1, σ_sim_ = 0.15 or σ_sim_ = 0.2 for the hydrostatic limit. The measurement for σ_sim_ = 0.1 has also been rescaled to match the expected experimental data, as described in the text.

Having illustrated that there is nothing special about the 4:1 ratio, we now seek to investigate further the role of EtOH in the mixtures. If its only role is to stall the crystallization of MeOH, then it follows that other liquids that could provide a similar hydrogen-bond environment should be equally effective. To this end, a 9:1 mixture of MeOH–*i*-PrOH was investigated experimentally. The 9:1 volume ratio was chosen for direct comparison with the 9:1 MeOH–EtOH ratio, which experimentally exhibited the highest hydrostatic limit. Fig. 6 ▸(*a*) shows the evolution of the standard deviation with uniaxial compression. Using the same criterion of σ = 0.1 defined previously, the hydrostatic limit of the 9:1 MeOH–*i*-PrOH mixture is 10.45 GPa, although a linear interpolation between the highest pressure point in the hydrostatic liquid (σ < 0.1) and the point just above (σ = 0.12) suggests that the hydrostatic limit (σ = 0.1) may fall even slightly above 11 GPa. As the value is very close to the hydrostatic limit for 9:1 MeOH–EtOH, the experiment suggests that *i*-PrOH is just as effective as EtOH at stalling the crystallization of MeOH without compromising the vitrification pressure.

Additional MeOH–*X* binary mixtures at a 9:1 volume ratio, where *X* = propan-2-ol, acetone, propanal, acetic acid, formic acid, acetonitrile, ethylene glycol, 1,2-dimethoxyethane, benzene, pyridine, dimethyl sulfoxide, octanol, cyclooctanol, tetrahydrofuran or *N*-methyl-2-pyrrolidone, chosen specifically to span molecule sizes, chemistry and hydrogen-bond propensity, were also investigated computationally. Fig. 6 ▸(*b*) shows the values of the theoretical hydrostatic limits obtained when using CGenFF and considering different σ_sim_ values as a threshold for hydrostaticity. The values obtained using σ_sim_ = 0.1 as the hydrostatic limit threshold have additionally been rescaled to match the experimental data via the same rescaling procedure described above. Following this, it is clear that the hydrostatic limits of all the binary mixtures fall within a very narrow range. The MeOH–EtOH mixture remains the one with the highest hydrostatic limit according to the σ_sim_ = 0.1 criterion, while all the other binary mixtures are within 1.5 GPa. A larger spread in hydrostatic limit was both observed and predicted for different volume ratios of MeOH–EtOH mixtures than was predicted for any of the binary mixtures at a 9:1 ratio. The results suggest that the quantity of MeOH in the mixture is much more influential on the vitrification pressure than the identity of the secondary component. The same conclusions can be drawn from the results obtained when using OPLS-AA, and a figure analogous to Fig. 6 ▸(*b*) can be found in the supporting information. Importantly, however, since MD simulations only capture vitrification rather than crystallization, they do not account for the effectiveness of secondary components with varying hydrogen-bonding abilities in delaying the crystallization of MeOH. Nevertheless, for secondary components with hydrogen-bonding characteristics similar to those of EtOH and *i*-PrOH, such as formic acid, acetic acid, ethylene glycol, octanol and cyclooctanol, all of which can function as both hydrogen-bond donors and acceptors, this opens the door to a broader range of potential PTMs with subtly distinct chemical behaviours.

## 4. Conclusions

MeOH–EtOH mixtures, along with the two pure components, have been investigated using both computational and experimental approaches. MD simulations reveal that the pressure at which hydrostatic conditions are lost increases monotonically with MeOH content, approaching the value observed for pure MeOH. Across all compositions studied, both at ambient pressure and under compression, no structural or dynamic property identified the commonly used 4:1 volume ratio as unique or exceptional.

Experimental results support this conclusion: the vitrification pressure increases consistently with MeOH content. Thus, as previously reported, EtOH appears to serve primarily to inhibit the crystallization of MeOH. We have shown experimentally that this is a role that is equally well performed by *i*-PrOH. Computational studies of a broader set of MeOH–*X* binary mixtures at a 9:1 volume ratio suggest that this behaviour extends beyond just MeOH–EtOH/*i*-PrOH systems. Mixtures with a wide range of secondary components demonstrate that the composition of this component has very little effect on the vitrification pressure. The requirement to stall the crystallization of MeOH probably restricts appropriate candidates further, but mixtures containing formic acid, acetic acid, ethylene glycol, octanol and cyclooctanol, which provide a similar hydrogen-bond competition, in a 9:1 ratio are promising candidates for PTMs with subtly different chemical behaviours.

Although the 4:1 MeOH–EtOH mixture is currently the most widely used PTM, our findings indicate that this ratio is not optimal but rather a by-product of the previous literature in the field, in conjunction with the limited exploration of the compositional space due to the time-consuming nature of the experiments. Higher hydrostatic limits can be achieved by increasing the MeOH content, provided a sufficient amount of a secondary component remains to inhibit crystallization. Notably, a 9:1 MeOH–EtOH mixture demonstrated an extension of nearly 1 GPa in the hydrostatic pressure range compared with the 4:1 MeOH–EtOH mixture, making it a promising and, crucially, readily available alternative PTM.

## 5. Related literature

For further literature related to the supporting information, see Frenkel & Smit (2002 ▸), Gowers & Carbone (2015 ▸), Hoover (1985 ▸), Jo *et al.* (2008 ▸), Kim *et al.* (2017 ▸), Lee *et al.* (2016 ▸), Lee *et al.* (2020 ▸) and Thompson *et al.* (2022 ▸). For the computational MD modelling, extensive use was made of the *LAMMPS* software (https://www.lammps.org/). Tools used to assist on generating initial configurations and to get CGenFF and OPLS-AA force field parameters to run the simulations in this work are available online at https://charmm-gui.org/, https://cgenff.com/ and https://trappe.oit.umn.edu/.

## Supplementary Material

Supporting information file. DOI: 10.1107/S1600576725008349/oc5048sup1.pdf

## Figures and Tables

**Table 1:** Hydrostatic limits of MeOH–EtOH mixtures and 9:1 MeOH–*i*-PrOH *P*_g_ represents the glass transition pressure and *P*_c_ represents the crystallization pressure. Results are presented for experimental (exp.) and simulated (sim.) data using the same standard deviation cut-off of 0.1. Simulated results are scaled to match the experimental data, as described in the text.

Composition	*P*_g_ sim. (GPa)	*P*_g_ sim. scaled (GPa)	*P*_g_ exp. (GPa)	*P*_c_ exp. (GPa)
EtOH	3.37	8.28	–	2.71
1:9	3.93	8.84	–	–
1:4	4.17	9.08	–	–
3:7	4.04	8.95	–	–
2:3	3.93	8.84	–	–
1:1	3.98	8.89	9.36	–
3:2	4.40	9.31	–	–
7:3	5.09	10.00	–	–
4:1	5.99	10.90	10.56	–
9:1	6.36	11.27	11.37	–
MeOH	5.82	10.73	–	6.70
9:1 MeOH–*i*-PrOH	5.79	10.70	10.45	–

## Data Availability

Supporting information for our work can be found at https://doi.org/10.5281/zenodo.16028744/. This includes (i) *LAMMPS* data files for all 11 MeOH–EtOH systems investigated in this work, containing potential parameters for the CGenFF, OPLS-AA, TraPPE and Guevara-Carrion *et al.* (2008 ▸) force fields; (ii) *LAMMPS* input files for the MD simulations at high pressure; (iii) in-house Python scripts written to compute hydrogen-bond lifetimes, to compute coordination numbers and to post-process the trajectories, as needed for computing PDFs for the centre of mass of the molecules; (iv) *LAMMPS* input scripts to compute the previously mentioned PDFs; (v) files containing the data used to generate the plots containing structural information and hydrogen-bond lifetimes at ambient pressure, and the self-diffusion coefficients, PDFs and densities at high pressure.

## References

[bb1] Angel, R. J., Bujak, M., Zhao, J., Gatta, G. D. & Jacobsen, S. D. (2007). *J. Appl. Cryst.* **40**, 26–32.

[bb2] Barnard, T. & Sosso, G. C. (2023). *J. Chem. Phys.* **159**, 014503. 10.1063/5.0156222 37403861

[bb3] Boehler, R. & De Hantsetters, K. (2004). *High Pressure Res.* **24**, 391–396.

[bb4] Bridgman, P. W. (1942). *Proc. Am. Acad. Arts Sci.* **74**, 399–424.

[bb5] Brugmans, M. J. P. & Vos, W. L. (1995). *J. Chem. Phys.* **103**, 2661–2669.

[bb6] Chen, B., Potoff, J. J. & Siepmann, J. I. (2001). *J. Phys. Chem. B* **105**, 3093–3104.

[bb7] Chervin, J., Canny, B. & Mancinelli, M. (2001). *High Pressure Res.* **21**, 305–314.

[bb8] Cook, R. L., Herbst, C. A. & King, H. E. Jr (1993). *J. Phys. Chem.* **97**, 2355–2361.

[bb9] Davies, H. W. (1968). *J. Res. Natl Bur. Stand. Sect. A* **72A**, 149. 10.6028/jres.072A.015 PMC6640600 31824085

[bb10] Eggert, J. H., Xu, L.-W., Che, R.-Z., Chen, L.-C. & Wang, J.-F. (1992). *J. Appl. Phys.* **72**, 2453–2461.

[bb11] Erdős, M., Geerdink, D. F., Martin-Calvo, A., Pidko, E. A., van den Broeke, L. J. P., Calero, S., Vlugt, T. J. H. & Moultos, O. A. (2021). *Appl. Mater. Interfaces* **13**, 8383–8394. 10.1021/acsami.0c20892 PMC7908017 33566563

[bb50] Frenkel, D. & Smit, B. (2002). *Understanding molecular simulation: from algorithms to applications.* Academic Press.

[bb12] Fujishiro, I., Piermarini, G. J., Block, S. & Munro, R. G. (1982). *High pressure in research and industry, 8th AIRAPT conference, 19th EHPRG conference, 17–22 August 1981, Institute of Physical Chemistry, University of Uppsala, Sweden, Proceedings*, pp. 608–611. Arkitektkopia.

[bb13] Gasol–Cardona, J., Ward, M. R., Gutowski, O., Drnec, J., Jandl, C., Stam, D., Maloney, A. G. P., Markl, D., Price, S. W. T. & Oswald, I. D. H. (2025). *Angew. Chem. Int. Ed.* **64**, e202412976. 10.1002/anie.202412976 PMC11720378 39545584

[bb14] Gelles, S. (1968). *J. Chem. Phys.* **48**, 526–527.

[bb51] Gowers, R. J. & Carbone, P. (2015). *J. Chem. Phys.* **142**, 224907–224917. 10.1063/1.4922445 26071731

[bb15] Groom, C. R., Bruno, I. J., Lightfoot, M. P. & Ward, S. C. (2016). *Struct. Sci. Cryst. Eng. Mater.* **72**, 171–179. 10.1107/S2052520616003954 PMC4822653 27048719

[bb16] Guevara-Carrion, G., Nieto-Draghi, C., Vrabec, J. & Hasse, H. (2008). *J. Phys. Chem. B* **112**, 16664–16674. 10.1021/jp805584d 19367909

[bb52] Hoover, W. G. (1985). *Phys. Rev. A* **31**, 1695–1697. 10.1103/physreva.31.1695 9895674

[bb17] Jayaraman, A. (1983). *Rev. Mod. Phys.* **55**, 65–108.

[bb53] Jo, S., Kim, T., Iyer, V. G. & Im, W. (2008). *J. Comput. Chem.* **29**, 1859–1865. 10.1002/jcc.20945 18351591

[bb18] Jorgensen, W. L., Maxwell, D. S. & Tirado-Rives, J. (1996). *J. Am. Chem. Soc.* **118**, 11225–11236.

[bb19] Katrusiak, A. (2008). *Acta Cryst.* A**64**, 135–148. 10.1107/S0108767307061181 18156679

[bb20] Katrusiak, A. (2019). *International tables for crystallography*, Vol. H, ch. 2.7. Chester: International Union of Crystallography.

[bb21] Keen, D. A. (2001). *J. Appl. Cryst.* **34**, 172–177.

[bb54] Kim, S., Lee, J., Jo, S., Brooks, C. L. III, Lee, H. S. & Im, W. (2017). *J. Comput. Chem.* **38**, 1879–1886. 10.1002/jcc.24829 PMC5488718 28497616

[bb22] Klotz, S., Chervin, J. C., Munsch, P. & Le Marchand, G. (2009). *J. Phys. D Appl. Phys.* **42**, 075413.

[bb55] Lee, J., Cheng, X., Jo, S., MacKerell, A. D., Klauda, J. B. & Im, W. (2016). *Biophys. J.* **110**, 641a.

[bb56] Lee, J., Hitzenberger, M., Rieger, M., Kern, N. R., Zacharias, M. & Im, W. (2020). *J. Chem. Phys.* **153**, 035103–035112. 10.1063/5.0012280 32716185

[bb23] Lewin, W. (2015). *8.01x – Lecture 27 – Fluid mechanics, hydrostatics, Pascal’s principle, atmospheric pressure*, https://www.youtube.com/watch?v=O_HQklhIlwQ.

[bb24] Li, Y., Feng, X., Liu, H., Hao, J., Redfern, S. A. T., Lei, W., Liu, D. & Ma, Y. (2018). *Nat. Commun.* **9**, 722. 10.1038/s41467-018-03200-4 PMC5818478 29459672

[bb25] Mammone, J., Sharma, S. & Nicol, M. (1980). *J. Phys. Chem.* **84**, 3130–3134.

[bb26] Maynard-Casely, H. E. (2017). *Crystallogr. Rev.* **23**, 74–117.

[bb27] Merrill, L. & Bassett, W. A. (1974). *Rev. Sci. Instrum.* **45**, 290–294.

[bb28] Moggach, S. A., Allan, D. R., Parsons, S. & Warren, J. E. (2008). *J. Appl. Cryst.* **41**, 249–251.

[bb29] Motaln, K., Uran, E., Giordano, N., Parsons, S. & Lozinšek, M. (2025). *J. Appl. Cryst.* **58**, 221–226. 10.1107/S1600576725000342 PMC11798517 39917187

[bb30] Oertel, H. (2004). *Prandtl’s essentials of fluid mechanics.* New York: Springer.

[bb31] Piermarini, G. J. (2001). *J. Res. Natl Inst. Stand. Technol.* **106**, 889. 10.6028/jres.106.045 PMC4865304 27500054

[bb32] Piermarini, G. J., Block, S. & Barnett, J. D. (1973). *J. Appl. Phys.* **44**, 5377–5382.

[bb33] Reeves, L. E., Scott, G. J. & Babb, S. E. Jr (1964). *J. Chem. Phys.* **40**, 3662–3666.

[bb34] Richards, R. Jr (2001). *Principles of solid mechanics.* Boca Raton: CRC Press.

[bb35] Schnabel, T., Srivastava, A., Vrabec, J. & Hasse, H. (2007). *J. Phys. Chem. B* **111**, 9871–9878. 10.1021/jp0720338 17661506

[bb36] Schnabel, T., Vrabec, J. & Hasse, H. (2005). *Fluid Phase Equilib.* **233**, 134–143.

[bb37] Shi, K., Smith, E. R., Santiso, E. E. & Gubbins, K. E. (2023). *J. Chem. Phys.* **158**, 040901–040933. 10.1063/5.0132487 36725519

[bb38] Shimizu, H., Nakamichi, Y. & Sasaki, S. (1990). *J. Raman Spectrosc.* **21**, 703–704.

[bb39] Soper, A. K. (2011). *Gudrun – routines for reducing total scattering data.* Science and Technology Facilities Council, Swindon, UK. https://www.isis.stfc.ac.uk/Pages/Gudrun.aspx.

[bb40] Sun, D., Minkov, V. S., Mozaffari, S., Sun, Y., Ma, Y., Chariton, S., Prakapenka, V. B., Eremets, M. I., Balicas, L. & Balakirev, F. F. (2021). *Nat. Commun.* **12**, 6863. 10.1038/s41467-021-26706-w PMC8617267 34824193

[bb41] Takemura, K. (2021). *High Pressure Res.* **41**, 155–174.

[bb57] Thompson, A. P., Aktulga, H. M., Berger, R., Bolintineanu, D. S., Brown, W. M., Crozier, P. S., in ’t Veld, P. J., Kohlmeyer, A., Moore, S. G., Nguyen, T. D., Shan, R., Stevens, M. J., Tranchida, J., Trott, C. & Plimpton, S. J. (2022). *Comput. Phys. Commun.* **271**, 108171–108205.

[bb42] Vanommeslaeghe, K., Hatcher, E., Acharya, C., Kundu, S., Zhong, J., Shim, J., Darian, E., Guvench, O., Lopes, P., Vorobyov, I. & Mackerell, A. D. Jr (2010). *J. Comput. Chem.* **31**, 671–690. 10.1002/jcc.21367 PMC2888302 19575467

[bb43] Wilson, C. J. G., Cervenka, T., Wood, P. A. & Parsons, S. (2022). *Cryst. Growth Des.* **22**, 2328–2341. 10.1021/acs.cgd.1c01427 PMC9007411 35431662

[bb44] Xu, M., Li, Y. & Ma, Y. (2022). *Chem. Sci.* **13**, 329–344. 10.1039/d1sc04239d PMC8729811 35126967

