# Supplementary material for: Optimal ratio or historical convention: the use of methanol–ethanol mixtures as pressure-transmitting mediums
Source: J Appl Crystallogr. 2025 Oct 24;58(Pt 6):1967–77. doi: 10.1107/S1600576725008349 (PMC12810705; doi:10.1107/S1600576725008349)
Supplement: Supplementary file 1 [file j-58-01967-sup1.pdf]

JOURNAL OF APPLIED CRYSTALLOGRAPHY

Volume 58 (2025)

Supporting information for article:

Optimal ratio or historical convention: the use of methanol–ethanol mixtures as pressure-transmitting media

Cameron J. G. Wilson, Cecilia M. S. Alvares, Anna Herlihy, Nicholas P. Funnell, Gabriele C. Sosso and Mark S. Senn

Optimal Ratio or Historical Convention: The Use of Methanol–Ethanol Mixtures as Pressure Transmitting Media.

Cameron J. G. Wilson [a], Cecilia M. S. Alvares [a], Anna Herlihy [b], Nicholas P. Funnell [c], Gabriele C. Sosso [a], and Mark S. Senn [a]

[a]Department of Chemistry, University of Warwick, Coventry CV4 7AL, United Kingdom
[b]Diamond Light Source, Harwell Campus, Oxfordshire, OX11 0DE, United Kingdom
[c]ISIS Neutron and Muon Facility, Rutherford Appleton Laboratory, Didcot, OX11 0QX, United Kingdom

1 Computational calculations

All molecular simulations performed in this work were made using LAMMPS version 29 Aug2024 (Thompson *et al.*, 2022). In the context of molecular dynamics simulations, the terminologies "NVT equations of motion" and "NPT equations of motion" are used to refer to the Nose-Hover equations of motion as originally formulated to sample the NVT and NPT ensembles, respectively (Hoover, 1985). Additionally, the Nose Hover equations of motion where only one dimension of the box is allowed to fluctuate are also used, with these being referred to as "NP_zT equations of motion" throughout the text (the length of the simulation domain in the z direction was the arbitrarily chosen one to fluctuate). In all these cases, the values of damping constants for the thermostat and (when applicable) barostat were, respectively, 100x and 1000x the value of the timestep used in the numerical integration, as advised elsewhere. (Developers, 2025*f*)

1.1 MeOH–EtOH: Molecular dynamics simulation details

Configurations for 1:9, 1:4, 3:7, 2:3, 1:1, 3:2, 7:3, 4:1, 9:1 volume ratios of MeOH–EtOH mixtures as well as for pure EtOH and MeOH were built using a python code to place the molecules in a box, later used as simulation domain. Information about atomic positions and connectivity for one methanol and ethanol molecule, necessary as input for the code, were obtained using charmm-gui (Im Lab, 2025; Jo *et al.*, 2008; Kim *et al.*, 2017; Lee *et al.*, 2016; Lee *et al.*, 2020). A random velocity generator was then used to assign velocities to the atoms. These microstates were used to start molecular dynamics simulations to study the systems at ambient temperature and pressure (300 K, 1 atm). For each system, four molecular dynamics simulations with the same setup were performed under ambient conditions. These four simulations differ in the force field used to model the interactions, which were CGenFF, OPLS-AA, TraPPE and the force field proposed by Guevara-

Carrion *et al* (2008) (referred throughout the text simply as Guevara-Carrion) (Vanommeslaedhe *et al.*, 2010; Jorgensen *et al.*, 1996; Chen *et al.*, 2001; Guevara-Carrion *et al.*, 2008; Schnabel *et al.*, 2007; Schnabel *et al.*, 2005). As the latter was parametrised specifically for ethanol and methanol, it is expected to better reproduce the properties of the system. Potential parameters in the case of CGenFF and TraPPE were obtained using tools available elsewhere, (Im Lab, 2025; Jo *et al.*, 2008; Kim *et al.*, 2017; Lee *et al.*, 2016; Lee *et al.*, 2020; SilcsBio, 2025; Group, 2025) while those for OPLS-AA and Guevara-Carrion were determined from the force fields' original publication (Jorgensen *et al.*, 1996; Guevara-Carrion *et al.*, 2008; Schnabel *et al.*, 2007; Schnabel *et al.*, 2005). A description of the molecular simulations made at ambient conditions and the corresponding properties obtained can be found in section 1.1.1. The microstate attained at the end of the production run at ambient conditions was then used as the initial microstate for the molecular dynamics simulations at high pressure for each of the 11 systems. At this stage, only the CGenFF and OPLS-AA force fields were considered. This is because the other two force fields feature constraints in bond lengths and/or 3-body-angles that would not be appropriate at high pressure. The specifics of the simulation setup at high pressure is common to the two force fields and is described in greater detail in section 1.1.2.

1.1.1 Setup: molecular dynamics simulations at ambient conditions

As the initial configurations for these simulations were artificially generated, an energy minimisation and some dynamics at low timesteps (0.01 fs and, subsequentially, 0.1 fs) using the NVT and/or NPT equations of motion were considered. In the latter case, the equations of motion contemplated target temperature and, when applicable, pressure of 100 K and 1 atm, respectively. Finally, the systems underwent an equilibration followed by a 1 ns long production run at target temperature and pressure of 300 K and 1 atm using the NPT equations of motion. At this stage, the timestep was raised to 1 fs and the equilibration length varied until instantaneous fluctuations of potential energy and volume around a constant mean value were reached. At all stages where NPT equations of motion were used, all dimensions of the box were allowed to vary, with the pressure control being coupled in the x, y and z directions.

Microstates were collected throughout the 1 ns production run to assess the properties of the systems. A total of 2000 configurations sequentially spaced by 500 fs were considered for computing pair distribution functions (g(r)), as defined within statistical mechanics, between all pairs of atoms (intramolecular pairs included). These functions were computed and output by LAMMPS considering a bin size of 0.01 Å (Developers, 2025*c*). These were then used for calculating the pair distribution function, G(r), as obtained in x-ray diffraction experiments (Keen, 2001). Note that this calculation was not performed for either the TraPPE and Guevara-Carrion force fields, as both prescribe implicit hydrogens - which prevent direct comparison with the experimental PDFs.

The same set of microstates used to compute the pair distribution functions were used to assess angle distribution functions (ADFs), continuous hydrogen bond life time (Gowers & Carbone, 2015)

and the coordination number of atoms involved in hydrogen bonds. The former was computed and output using LAMMPS, (Developers, 2025a; Developers, 2025d) while the two latter were calculated using in-house python codes, which can be found at (Zenodo, 2025). The ADFs calculated concern the angles O-H$\cdots$O. Only the closest neighbouring intermolecular H$\cdots$O pairs were considered in the calculation. A suitable distance criteria to target only the closest pairs can be determined using the pair distribution functions built for intermolecular hydrogen-oxygen pairs. In order to assess all possible hydrogen bonds that can form in the binary mixture, MeOH and EtOH need to be considered both as a hydrogen bond donor and acceptor. The normalisation of the ADFs (the normalisation here implies a summation to one) was done by LAMMPS. It is worth noting that the first H$\cdots$O neighbours in all systems within all force fields sit at a distance smaller than 3 Å, which is considered as a distance criterion for a hydrogen-oxygen pair to be hydrogen bonded (Gowers & Carbone, 2015). Additionally considering that the O-H$\cdots$O angle needs to be larger or equal to 130° for a hydrogen bond to exist, (Gowers & Carbone, 2015) these ADFs ultimately provide the fraction of hydrogen bonds in a system.

For computing the continuous hydrogen bond lifetime, 100 groups of 200 configurations (or subtrajectories) consecutively spaced by 500 fs from each other were created from the 2000 configurations collected during the production run were considered. These groups, which naturally share some configurations, feature, each, a different initial configuration. First, all H$\cdots$O pairs in the simulation domain that can potentially form hydrogen bonds are considered and labelled by the IDs of the atoms forming the given pair, ij. Then, for the initial configuration of each subtrajectory and using the hydrogen bond criteria described above, the pairs that effectively are found to be hydrogen bonded are identified and distinguished from those which are not. This is mathematically translated into a column vector where each line correspond to specific pair of hydrogen and oxygen atoms, ij, in the simulation domain and elements 1 and 0 used to distinguish those which are hydrogen bonded (1) and those that are not (0). Subsequently, all other configurations belonging to this subtrajectory were consecutively assessed to see if each of the initial hydrogen bonds still exist. A 1D array having the same length, line-to-atom-pair-correspondence and meaning as for the initial configuration is built and values of 0 were assigned to the hydrogen bonds that have been broken. Since the calculation aims to determine continuos hydrogen bond lifetime, once broken, hydrogen bonds were no longer considered even if formed again. Since each subtrajectory has 200 configurations consecutively spaced by 500 fs and thus span for a total of 1 ps, information on how many of the hydrogen bonds that existed at $t = 0$ still remains can be found for any time instant that is multiple of 500 fs up to a value of 1 ps. Once this is calculated for all configurations in a subtrajectory and for all subtrajectories, the function $C(t)$, given in equation 1, can be computed. $C(t)$ is the time autocorrelation function, h symbolises the 1D array previously mentioned and ij represent a specific line of the column vector and thus a given hydrogen and oxygen pair that may be potentially hydrogen bonded in the simulation domain. In the numerator of the expression inside the brackets lies, at a given arbitrary time t, the summation of the product of the value stored in the same lines of the column vectors built at time $t = 0$ and at the time t for a given

subtrajectory. Given the 0 and 1 convention previously mentioned, this summation corresponds to the number of hydrogen bonds that existed at time $t = 0$ that still remain at time t within the given subtrajectory. The denominator on the other hand corresponds simply to the sum of hydrogen bonds that exist at time $t = 0$ in that same subtrajectory, meaning the division yields the fraction of hydrogen bonds that remain, from the ones at time $t = 0$, after a given amount of time has passed. The averaging denoted by the brackets refers, at each time instant, to the mean value computed over the 100 subtrajectories included in the analysis. Once the function $C(t)$ is determined, the continuous hydrogen bond lifetime can be determined. It is defined as the integral $\int_{t=0}^{\infty} C(t)$. notably, the limit of $C(t)$ as $t \to \infty$ should be 0. Since the values of $C(t = 100ps)$ are already relatively small for all systems and force fields, the upper limit of the integral previously mentioned was replaced by $t = 100$ ps in all cases to compute the hydrogen bond lifetime.

$$C(t) = \left\langle \frac{\sum (h_{ij}(t=0)h_{ij}(t))}{\sum h_{ij}^2(t=0)} \right\rangle \tag{1}$$

To assess the coordination number of the oxygen atoms involved in hydrogen bonds, all oxygen atoms in the system were analysed and categorised accordingly into a "hydrogen bond scenario". Hydrogen bonds are considered to exist based on the same distance and O-H···O angle thresholds mentioned above. In the mixtures, this assessment is made individually for oxygens of methanol and ethanol molecules, and a distinction is made on whether the hydrogen atom belongs to a methanol or ethanol molecule. Seven different "hydrogen bond scenarios" are considered depending on how many hydrogens are found: (i) no H, (ii) 1 H_{MeOH}, (iii) 1 H_{EtOH}, (iv) 2 H_{MeOH}, (v) 2 H_{EtOH}, (vi) 1 H_{MeOH} and 1 H_{EtOH}, (vii) none of the previous alternatives. A maximum of two hydrogen bonds per oxygen is expected to occur, a scenario where more than two Hs are counted is expected to stem from low probable events of having a third hydrogen lying within the bond and angle threshold setup but not actually hydrogen bonded to the given oxygen. The information is organised in the form of a histogram, in which the x-axis features different possible "hydrogen bond scenarios" and the y-axis informs the number of oxygens meeting the given scenario within all configurations considered in the calculation. The histograms are normalised by the number of oxygens in the given system and the number of configurations used in the calculation. This normalisation is necessary in order to compare histograms for different systems as these have a different number of molecules of each compound.

Finally, the calculation of pair distribution functions for intermolecular oxygen and hydrogen pairs capable of hydrogen bonding was also made using LAMMPS and considering the 2000 configurations saved in the production run (Developers, 2025c; Developers, 2025d). These functions were built individually for all distinct O and H pairs. Additionally, pair distribution functions for MeOH-MeOH, MeOH-EtOH and EtOH-EtOH molecule pairs were also computed in order to assess the structure in a coarser way. Firstly, a coarse trajectory in LAMMPS format was obtained out of the 2000 configurations collected during the production run. This is accomplished using LAMMPS commands (Developers, 2025b; Developers, 2025d) interfaced with a simple python code written

in-house. This coarse trajectory contains, for each configuration, the information about the position of the centre of mass of all methanol and ethanol molecules that compose a given system, which, for the sake of the LAMMPS formatting, are assigned each a different ”atom ID”. Additionally, methanol and ethanol molecules in this coarse trajectory are distinguished by their ”atom type” (1 and 2 are used for MeOH and EtOH molecules, respectively). Pair distribution functions are then calculated using LAMMPS. All the LAMMPS input scripts and the python code used in the process can be found elsewhere (Zenodo, 2025).

1.1.2 Setup: molecular dynamics simulations at high pressures

To study the systems under uniaxial compression, NP_zT equations of motion are used to carry out the dynamics of the atoms using a 1 fs timestep. The target temperature considered in these equations is always set to be 300 K, while, starting from a value of 0.2 GPa, the target P_{zz} value is ”abruptly” increased by 0.2 GPa every 2 ns up to a maximum value of 12 GPa (no pressure ramp is considered). As mentioned in the main text, microstates stemming from the last 1 ns of the dynamics at each target P_{zz} were used to estimate the properties of the system.

The self diffusion coefficients for MeOH and EtOH molecules at each P_{zz} were estimated considering a set of 2000 configurations attained every 500 fs in the last 1 ns of the dynamics at the given target P_{zz} value using the relationship between time (t) and mean-square displacement (MSD). These 2000 configurations were used to form 100 different groups (or subtrajectories). Each group is formed by 1000 consecutive configurations contemplating the regular spacing of 500 fs, and thus spanning a total of 0.5 ns of simulation time each. The starting configurations for each group are consecutively spaced by 5000 fs. The mean square displacement of the molecules were calculated for each group of configurations, thus yielding 100 different sets of t, MSD data. This calculation was made using LAMMPS and, in the case of the MeOH–EtOH mixtures, the mean square displacement of methanol and ethanol molecules were calculated separately (Developers, 2025e; Developers, 2025d). The 100 values of MSD at each t were averaged and then used to compute the self diffusion coefficients by fitting a linear function to $MSD = f(t)$, expected at high t values (Frenkel & Smit, 2002). For the sake of completion, the calculation was also made at ambient conditions in the simulations where OPLS-AA and CGenFF are used, so that self diffusion coefficient covers the entire pressure range studied here. Furthermore, it is important to note that as pressure increases and translational motion is more difficult, longer simulations are necessary in order to properly attain the relationship $MSD(t)$ that holds at long time instants. As a result, the self diffusion coefficient values at high P_{zz} values obtained herein should be interpreted with caution.

A representative number of configurations (10^{3-4}) collected during the production were used to compute the pair distribution functions for all atom pairs. This calculation was performed using LAMMPS (Developers, 2025c). Similar to the assessment of the structure at ambient conditions (see section 1.1.1), the pair distribution functions output by LAMMPS were then used to compute

the G(r)-analog of that obtained from x-ray diffraction experiments (Keen, 2001). This calculation was made only for 3:2, 7:3, 4:1 and 9:1 MeOH–EtOH mixtures, as the goal was to specifically spot any outlying behaviour, from the structural point of view, for the 4:1 volume fraction from its neighbouring compositions. Furthermore, only G(r)s at P_{zz} of 4 GPa, 5 GPa, 6 GPa and 7 GPa were computed, aiming to focus the analysis near the onset of the loss of hydrostaticity observed for these systems. Differently from what was made at ambient condition, the calculation excluded 1-2, 1-3 and 1-4 neighbours.

Finally, the instantaneous values of P_{xx}, P_{yy} and simulation domain volume attained every 100 fs during the last 1 ns of the dynamics at each target P_{zz} value were computed and output by LAMMPS. These were then split into 5 groups of 2000 values and for each group an average value of P_{xx}, P_{yy} and volume of the simulation domain was calculated. The observables and their standard error were ultimately estimated from these five average values. The values of P_{xx} and P_{yy} were used to estimate the value of σ_{sim}, defined in main text, while the volume of the simulation domain was used to estimate the density under different degrees of uniaxial compression. Aiming to have density data throughout the entire pressure range investigated in our work for CGenFF and OPLS-AA, the volume of the simulation domain was also computed at ambient pressure for completion, with the density being estimated following the same setup previously described.

1.2 MeOH–EtOH: Molecular dynamics simulations' extra results

1.2.1 Ambient temperature and pressure

The figures in this section show the results obtained for pure MeOH, pure EtOH and their mixtures at ambient conditions (300 K, 1 atm) when using each of the four force fields considered in this work to model the interactions. Figure S1 shows the fraction of oxygens in the system that can be found to be hydrogen-bonded to a given number of hydrogens on average. Figure S2 shows the continuous hydrogen bond lifetime, which corresponds to average time a hydrogen and oxygen pair remains hydrogen bonded in the systems (without accounting for hydrogen bond reformation). Figures S3 and 4 show the pair distribution functions built for different molecules (centre of mass based) and for intermolecular oxygen-hydrogen pairs, respectively. Figure S5 shows the ADFs for oxygen-hydrogen-oxygen angles where one of the oxygens is chemically bonded to the given hydrogen (central atom) and the other oxygen lies within a distance of 3 Å from it.

While the force fields predict slightly different lifetimes to one another for each given hydrogen bond, they follow the same trend: the lifetime decreases as the system becomes richer in MeOH. This aligns with the faster diffusion of both MeOH and EtOH molecules observed as the MeOH fraction increases. Small bumps associated with O-$H_M\cdots$O and O-$H_E\cdots$O hydrogen bonds in the region of low MeOH and low EtOH content, respectively, is believed to be associated with random error rather than something physically meaningful. A smooth and consistent trend is observed across all previously discussed properties, with MeOH–EtOH mixtures gradually approaching the behaviour of pure MeOH or pure EtOH as their respective concentrations increase. The only exception is

the ADFs, which appear similar across all compositions. Ultimately, this suggests that, within the scope of the four force fields used to model the interactions, no anomalous structural behaviour emerges at any specific composition under ambient conditions.

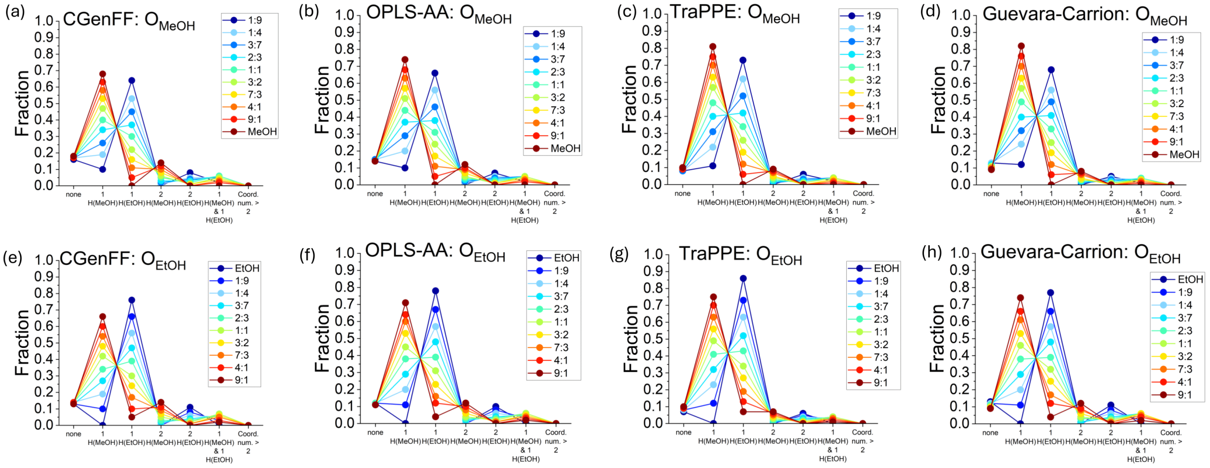

Figure S 1: Fraction of occurrence of the different "hydrogen bond scenarios" experienced by the O_{MeOH} (upper panels) and O_{EtOH} (lower panels) at 300 K and 1 atm according to each of the four force fields considered to model the interactions

Analogously to the figure presented in the main text for CGenFF, figure S6 show the pair distribution functions built for pure EtOH, pure MeOH and some of its mixtures obtained in the simulations when using the OPLS-AA force field overlapped with the experimental counterpart.

1.2.2 Ambient temperature and uniaxial compression

Figure 7 displays the evolution of density with uniaxial compression for pure MeOH, pure EtOH and their mixtures when interactions are modelled using CGenFF or OPLS-AA. No unexpected trends for any specific composition can be identified in these plots, consistently with the conclusion made in the main text about the unimportance of a specific volume ratio. In addition to the discussion in the main text, figure 8 shows the hydrostatic limit for these systems if considering σ_{sim} equal to 0.15 or 0.2 as a criteria for loss of hydrostaticity when interactions are modelled using CGenFF. Results obtained upon using the same scaling as discussed in the main text (i.e. summation by 4.91 GPa) is also shown. Finally, figure 9 shows results analogous to those presented in the main text for CGenFF at high pressure for MeOH–EtOH systems when the interactions are modelled using OPLS-AA. These are the the evolution of σ_{sim}, D_{MeOH} and D_{EtOH} with P_{zz}, the hydrostatic limits (including values scaled by adding a value of 4.91 GPa, as done for CGenFF in the main text) observed upon using different values of σ_{sim} as criteria and the G(r) of the 3:2, 7:3, 4:1 and 9:1 MeOH–EtOH mixtures at 4-7 GPa.

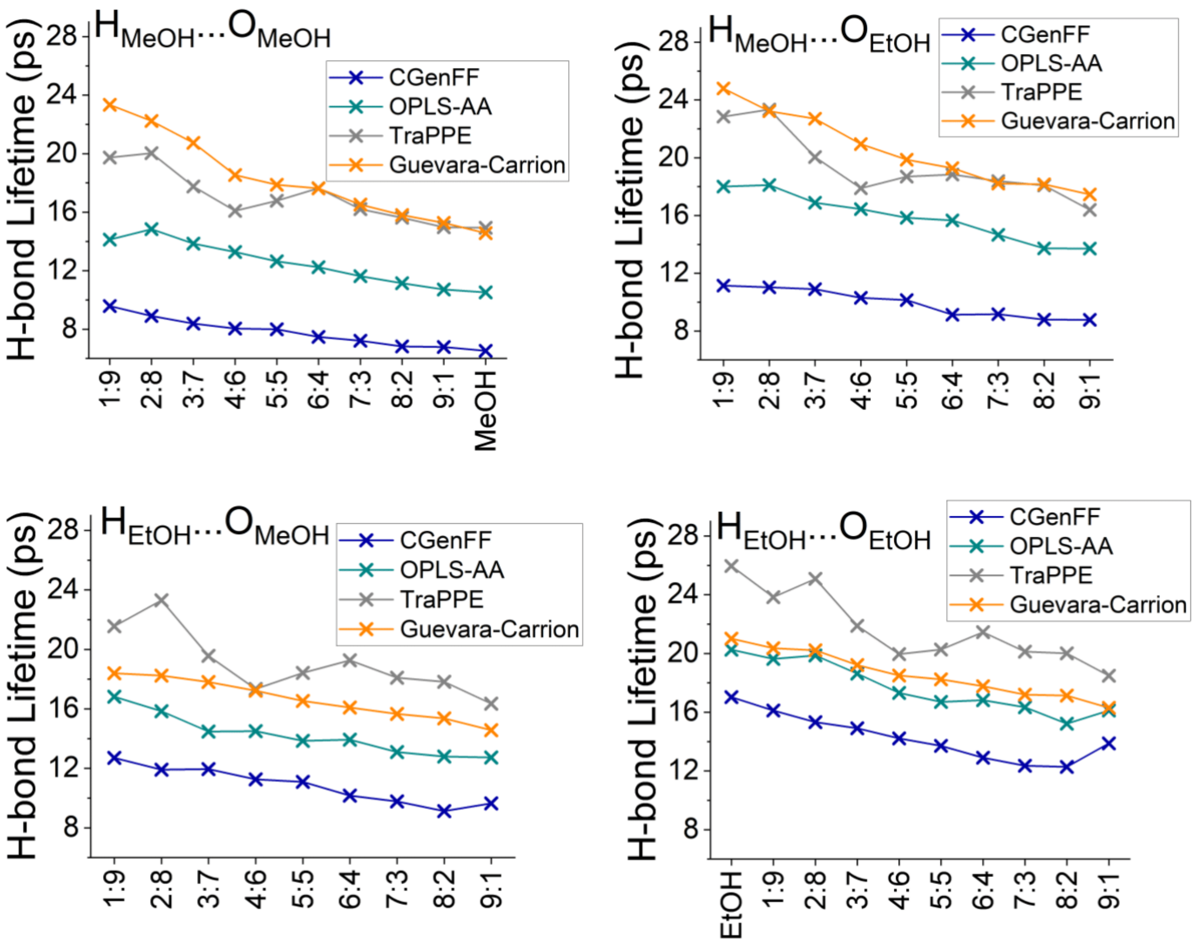

Figure S 2: Continuous hydrogen bond lifetime values predicted by each of the four force fields considered in this work for the (a) $H_{MeOH}\cdots O_{MeOH}$, (b) $H_{MeOH}\cdots O_{EtOH}$, (c) $H_{EtOH}\cdots O_{EtOH}$, (d) $H_{EtOH}\cdots O_{EtOH}$ hydrogen bonds found in each applicable system at 300 K and 1 atm. In the labels of the x-axis, "ME" is used as an abbreviation of "MeOH–EtOH" for clarity.

1.3 9:1 MeOH–X mixtures: OPLS-AA results

Figure 10 shows the hydrostatic limit obtained for all 9:1 volume ratio MeOH–X mixtures, where X = 2-propanol, acetone, propanal, acetic acid, formic acid, acetonitrile, ethylene glycol, 1-2-dimethoxyethane, benzene, pyridine, dimethyl sulfoxide, octanol, cyclooctanol, tetrahydrofuran or N-methyl-2-pyrrolidone, when the interactions are modelled using the OPLS-AA force field. In analogy to what is done when using CGenFF to model the interactions, values of 0.1, 0.15 and 0.2 for σ_{sim} are used as thresholds for the hydrostatic limit, with the values in the former case being scaled by 4.19 GPa to match the ones obtained experimentally.

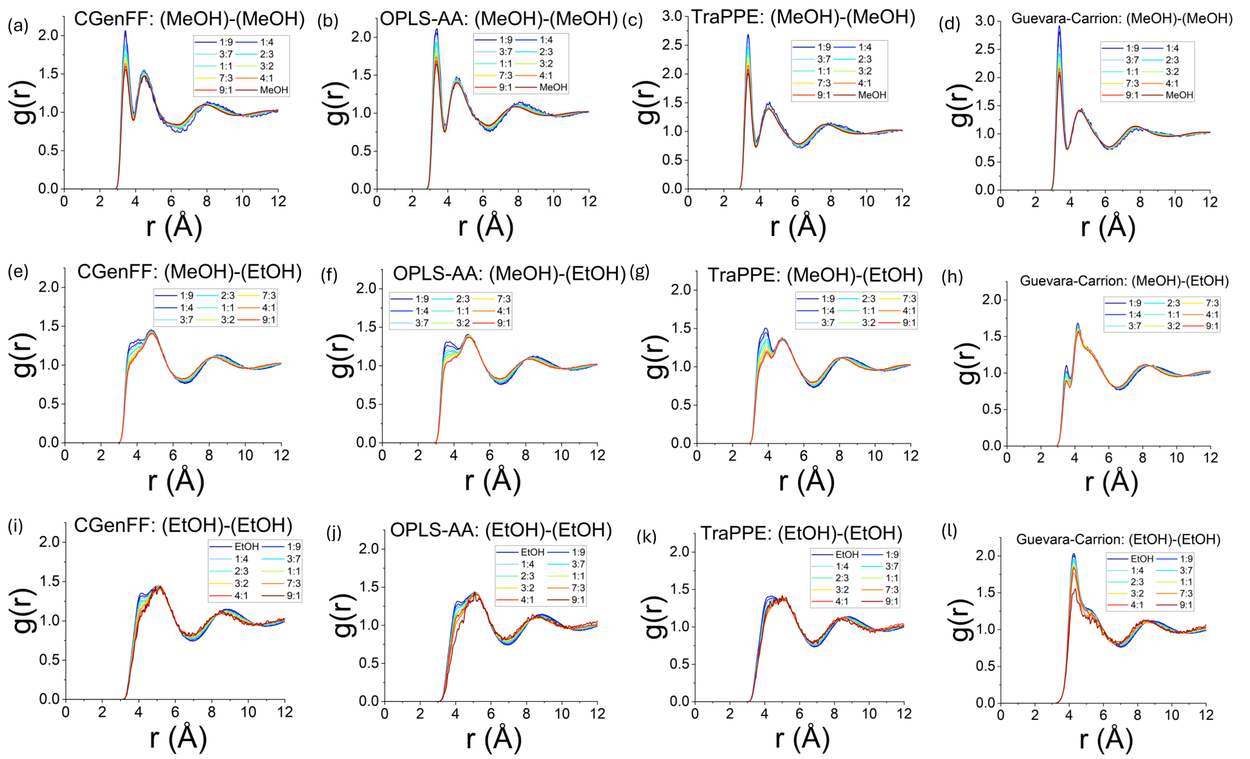

Figure S 3: Pair distribution functions for (MeOH)-(MeOH), (MeOH)-(EtOH), (EtOH)-(EtOH) molecules. The first, second, third and fourth columns correspond to the functions obtained for each system at 300 K and 1 atm when modelling the interactions using the CGenFF, OPLS-AA, TraPPE and the Guevara-Carrion force fields.

2 Experimental

For every high-pressure experiment, 7 rubies were loaded into the sample chamber. These were distributed as evenly as possible with a minimum amount of high vacuum grease. Attempts were intentionally made to provide a range of ruby sizes ($\leq$ 50 μm) in each loading. Images of the sample chambers are presented in the main text with the exception of the repeat loading of 9:1 MeOH–EtOH which is presented in Figure S11 below. Pressure measurements were completed with an Almax easyLab Optiprexx PLS (photoluminescence) device, a stand-alone spectrometer featuring a 532 nm, 50 mW laser. For each ruby measurement, the laser was first optically centred on the ruby being measured and the signal then maximised by fine adjustments to account for inaccuracies in the optical set up. In early investigations where peak width was also mapped to follow the loss of hydrostaticity (before this was found to be less accurate than following the standard deviation in the ruby pressures, similar to (Klotz *et al.*, 2009) and (Motaln *et al.*, 2025)) a unique integration time was optimised for each ruby and then held constant throughout each PTM study. In subsequent experiments integration time and scan averaging was further optimised to produce the best signal to noise ratio possible for each measurement. Pressure was increased mechanically in the smallest steps possible. After pressure was increased, a minimum wait time of

15 mins was allowed to pass before the pressure of the first ruby was measured. Rubies were then measured in turn in a consistent order until repeated measurements of the same ruby in subsequent cycles gave the same pressure measurement. These results were then recorded. The pressure of the rubies were then measured in a similar fashion at regular intervals (0.5-1 hours) until the pressure of all rubies was stable for a minimum of two hours. The pressure was then mechanically increased again. Data presented for each PTM were from a single loading only with the exception of the 9:1 mixture where a secondary loading was completed to alleviate concerns with the spacing of points near the hydrostatic limit. Rubies and gaskets were not reused between experiments.

The pressure of each ruby in the first (black triangles in the main text) 9:1 MeOH–EtOH loading vs the average pressure is provided in Figure S12. The distribution of pressures increases at the reported hydrostatic limit of 11.37 GPa. The spread is relatively symmetric and evidences that the loss in hydrostaticity is a global event rather than the degradation of an individual ruby. There was no statistical trend between the size of the ruby and the deviation from the average pressure. The standard deviation of the pressures was calculated as in (Klotz *et al.*, 2009) using the following equation

$$\sigma = \sqrt{\frac{1}{N}\sum_{i=1}^{N}(P_i - \bar{P})^2} \tag{2}$$

where P_i is the pressure of ruby i, $\bar{P}$ is the average pressure (arithmetic mean) and N is the number of rubies (always 7 in this instance).

Within the manuscript, all experimental hydrostatic limit plots are presented on the same axes for ease of comparison. For MeOH this means the highest pressure points are beyond the bounds of the axes. All pressure points for MeOH are presented in Figure S13.

2.1 The Stability of 9:1 MeOH–EtOH

To alleviate concerns with respect to the long-term stability of the 9:1 MeOH–EtOH mixture, additional experiments have been completed. The first of these pertained to the storage of a 9:1 and 4:1 mixture of MeOH–EtOH in the fridge for two weeks. There were no perceivable differences in either mixture during this time, Figure S14. It should be noted that premixed solutions should not be stored for much longer than this because of the vulnerability of MeOH to uptake of water.

Secondly, the stability of the solution at pressure was tested. Figure S15 shows the sample chamber within a DAC loaded with 9:1 MeOH–EtOH before and after two weeks at pressures higher than the crystallisation pressure of both MeOH and EtOH as determined in this work. It is clear from this image that the solution is not crystallised and does not resemble the images in Figure 3 in the main text. A small deformation of the gasket hole is observed, which is not uncommon for high-pressure experiments. Together, these results indicate that the 9:1 solution is suitable for use for extended periods of time at lower temperatures and at high pressures.

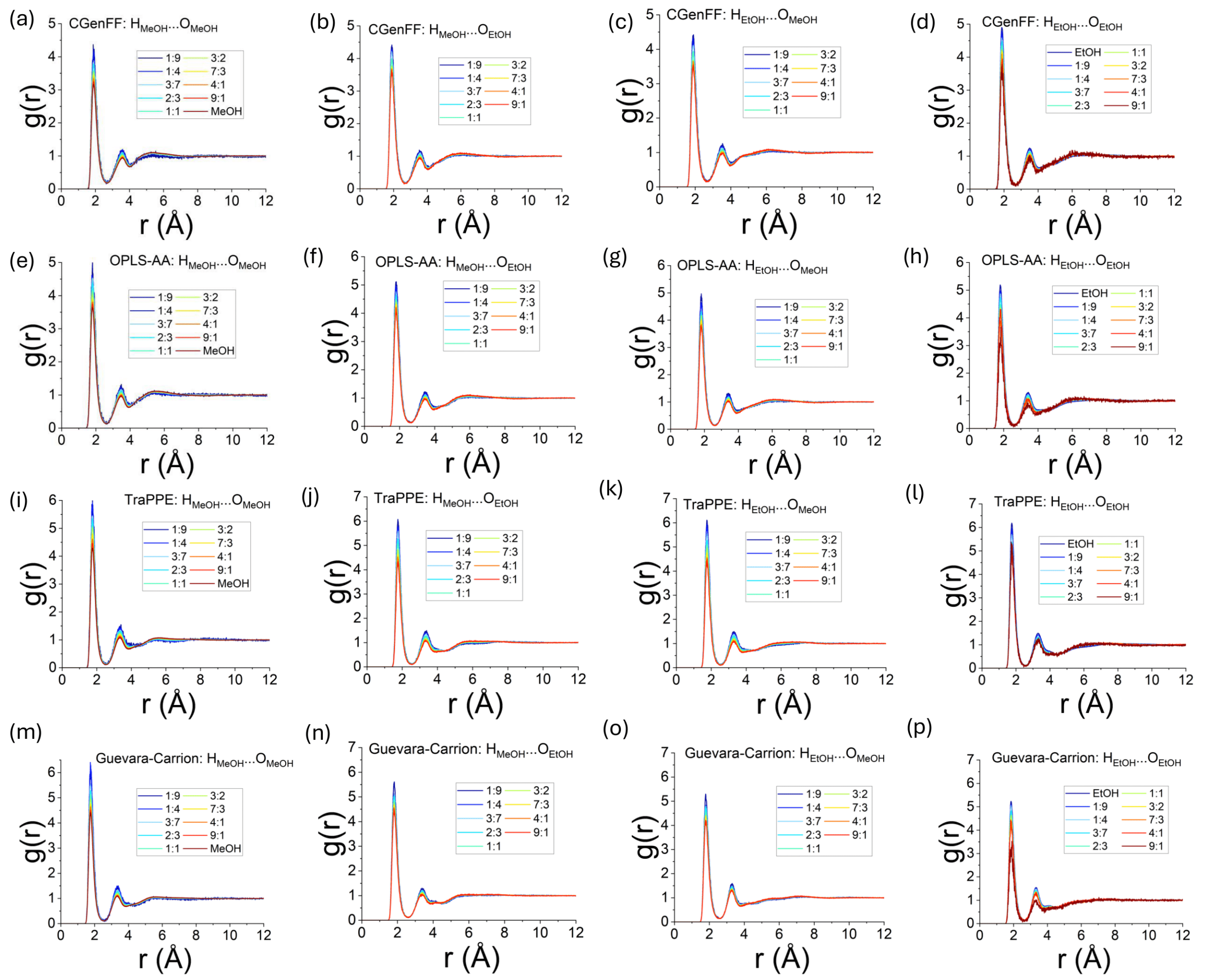

Figure S 4: Pair distribution functions for all distinct intermolecular oxygen-hydrogen atom pairs that can exist within the systems. Specifically, the first, second, third and fourth columns in the figures correspond to the pairs $H_{MeOH} \cdots O_{MeOH}$, $H_{MeOH} \cdots O_{EtOH}$, $H_{EtOH} \cdots O_{MeOH}$, $H_{EtOH} \cdots O_{EtOH}$, respectively. Plots for each due system obtained using all four force fields considered in this work are presented.

,

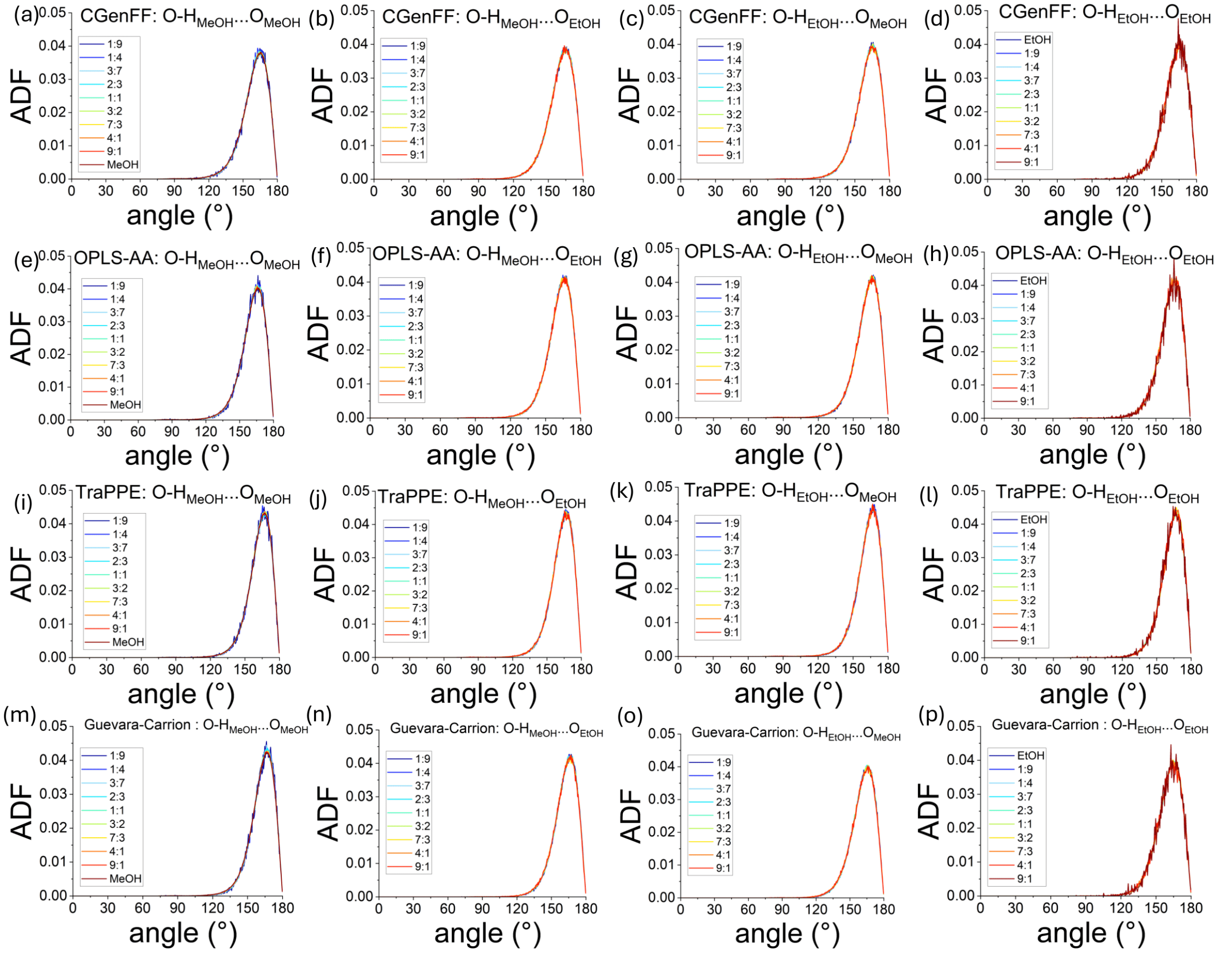

Figure S 5: Angular distribution function for the O-H$\cdots$O angles in the system where the H$\cdots$O pair lie in the first coordination shell of one another. Histograms for the angles featured in all the "types" of hydrogen bonds that can occur in MeOH–EtOH binary mixtures are shown. Specifically, the first, second, third and fourth columns show plots for the 3-body angles O_{MeOH}-$H_{MeOH}\cdots O_{MeOH}$, O_{MeOH}-$H_{MeOH}\cdots O_{EtOH}$, O_{EtOH}-$H_{EtOH}\cdots O_{MeOH}$, O_{EtOH}-$H_{EtOH}\cdots O_{EtOH}$, respectively. Plots for each due system obtained using all four force fields considered in this work are presented.

,

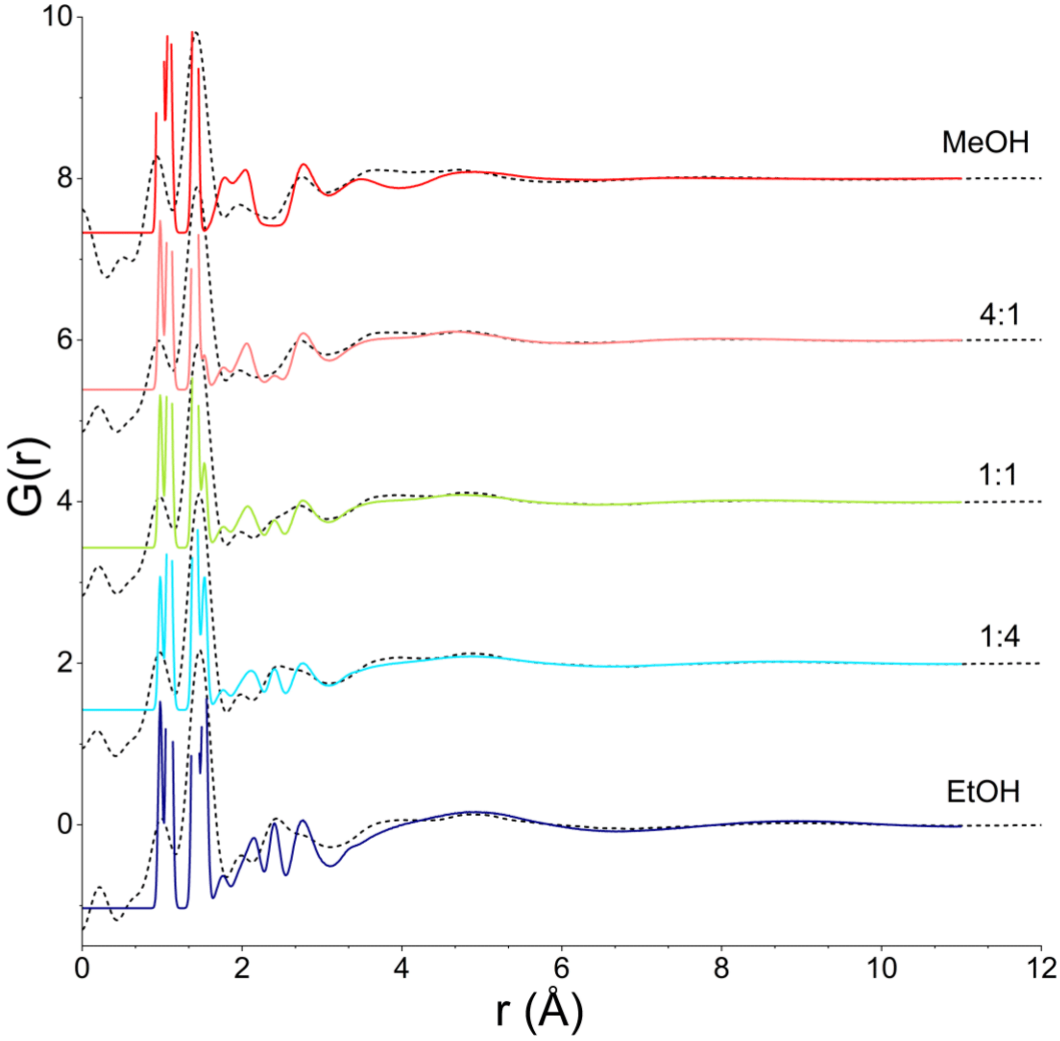

Figure S 6: Pair distribution function at ambient temperature and pressure for pure MeOH, pure EtOH, 1:4, 1:1 and 4:1 volume ratio MeOH–EtOH mixtures (coloured solid lines) overlapped with the due experimental PDF obtained in x-ray diffraction experiments (black dashed lines). Curves for different compositions were offset for clarity.

,

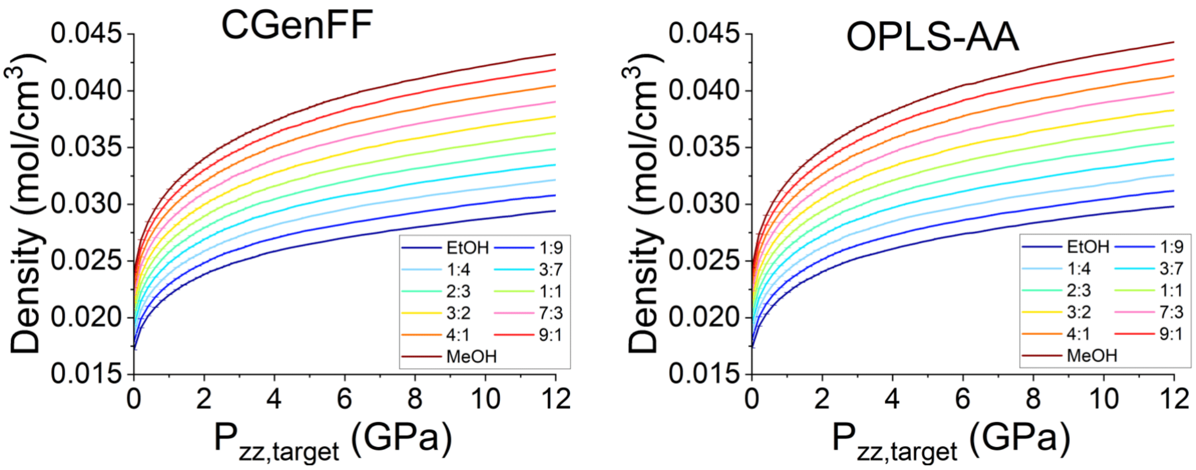

Figure S 7: Density in $mol\,cm^{-3}$ for pure MeOH, pure EtOH and their binary mixtures at different pressures when the interactions are modelled using (a) CGenFF and (b) OPLS-AA

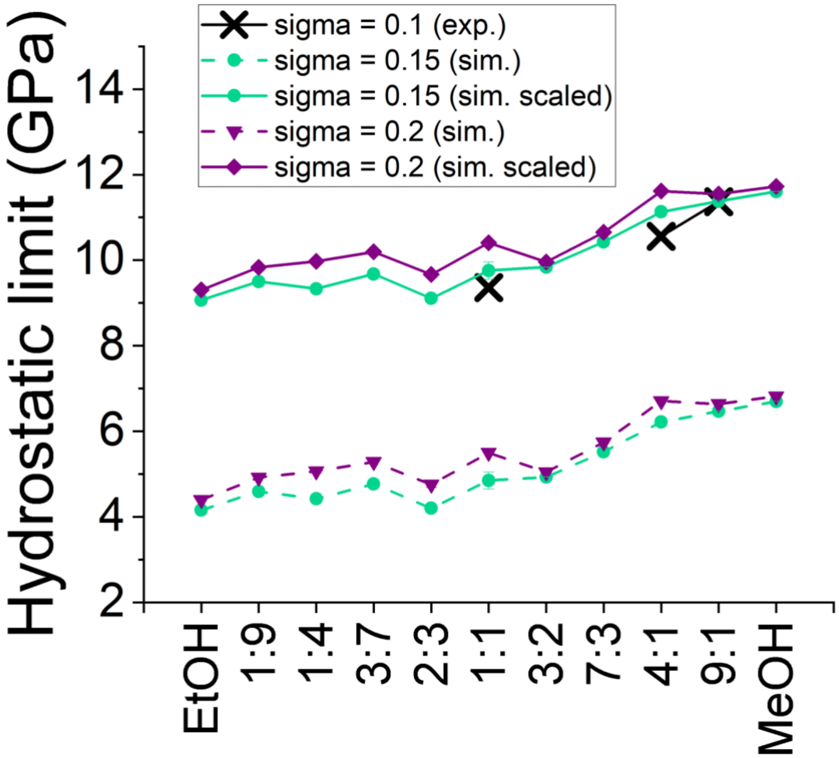

Figure S 8: Hydrostatic limits obtained for pure MeOH, pure EtOH and their mixtures when using CGenFF to model the interactions together with the values obtained in our experiments when available. Criteria $\sigma_{sim} = 0.15$ and $\sigma_{sim} = 0.2$ are considered for loss of hydrostaticity. Curves obtained by summing 4.91 GPa to the hydrostatic limit are also shown.

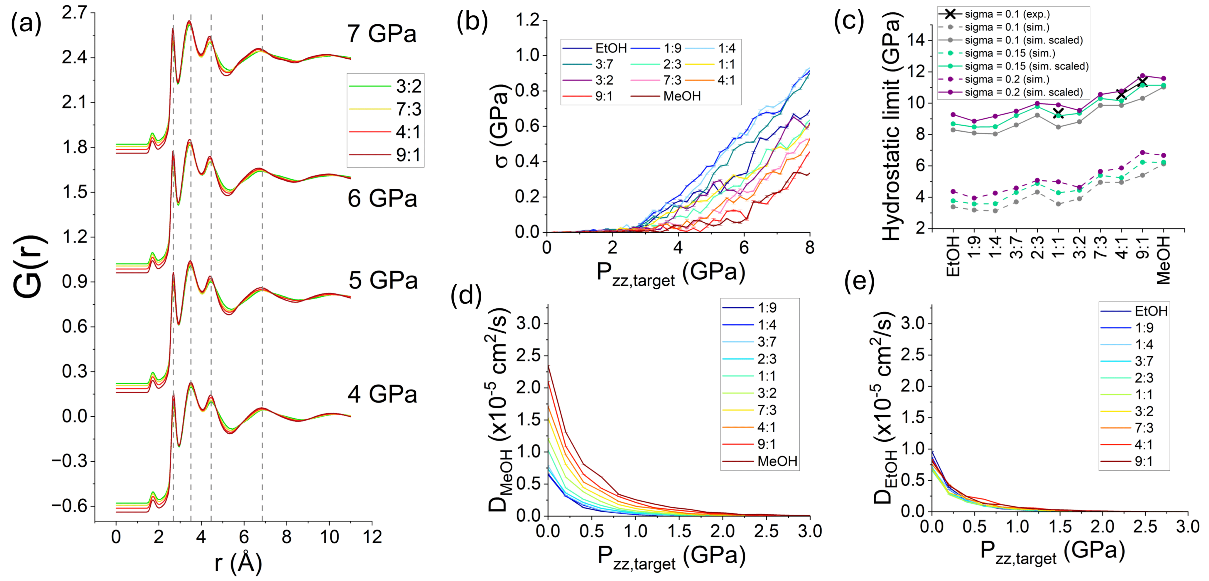

Figure S 9: (a) Simulated pair distribution functions obtained for different MeOH–EtOH mixtures at different degrees of uniaxial compression; evolution of (b) σ_{sim} with compression; (c) values of hydrostatic limit obtained for the different MeOH–EtOH systems investigated using different values of σ_{sim} as criteria (gray, green and purple lines) along with values observed experimentally for systems studied (black crosses); evolution of (d) D_{MeOH} and (e) D_{EtOH}, respectively, with compression. In pannel (a), gray vertical dashed lines are presented to assist visualisation of peak mode shifting relative to what is observed at $P_{zz} = 4$ GPa. In pannel (c), dashed lines are used for the values originally obtained upon using the different values of σ_{sim} as criteria while solid lines add to them the same scaling (4.91 GPa) as used for CGenFF in the manuscript.

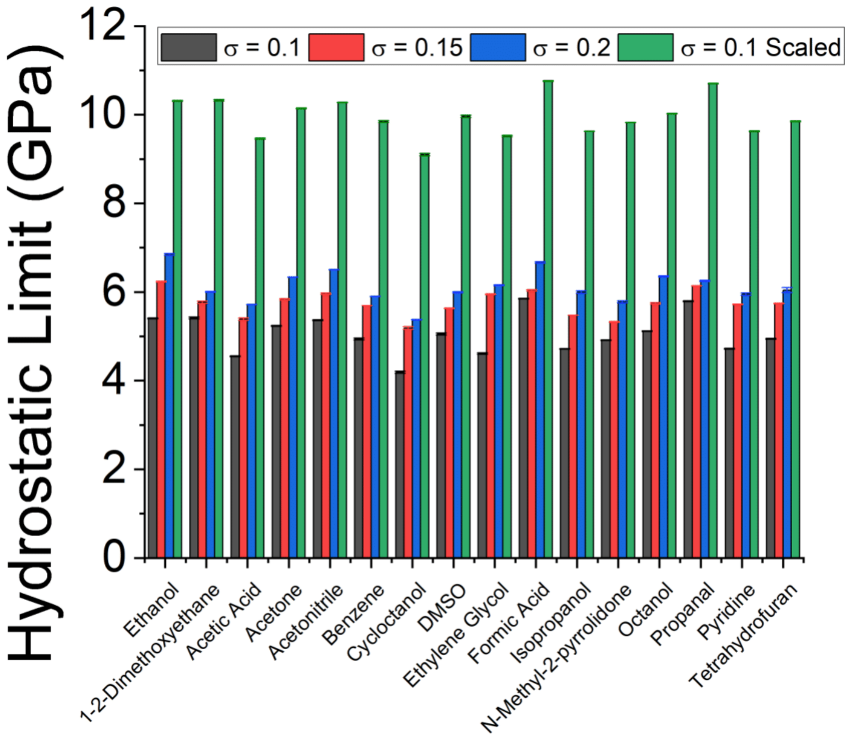

Figure S 10: Hydrostatic limit obtained for the different 9:1 MeOH-based mixtures investigated in our work when the OPLS-AA force field is used to model the interactions. Different σ_{sim} values are used as criterion for the end of hydrostaticity. The values obtained upon considering $\sigma_{sim} = 1$ as criterion were scalled by adding 4.19 GPa, as done in the main text.

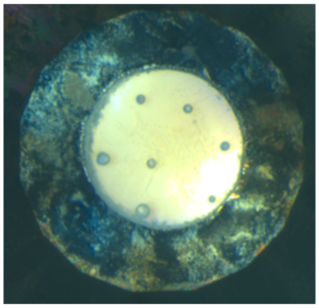

Figure S 11: Pressure chamber of second 9:1 MeOH-EtOH loading.

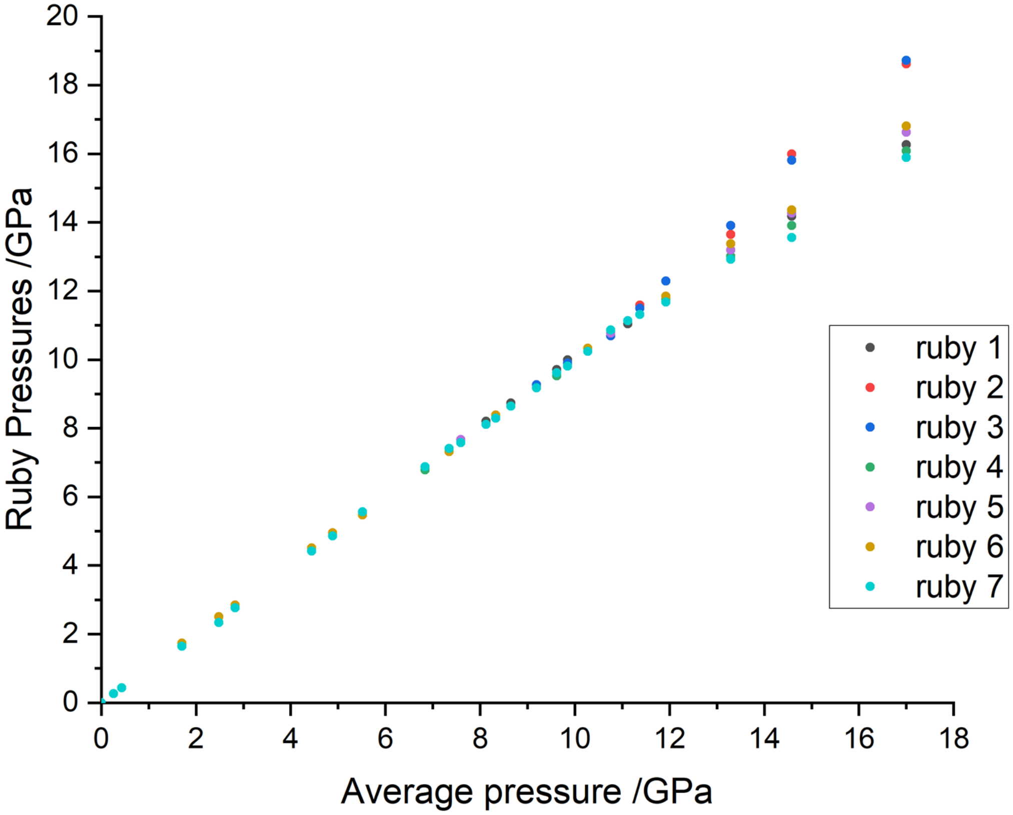

Figure S 12: The spread of pressures from individual rubies as average pressure increases for 9:1 MeOH–EtOH.

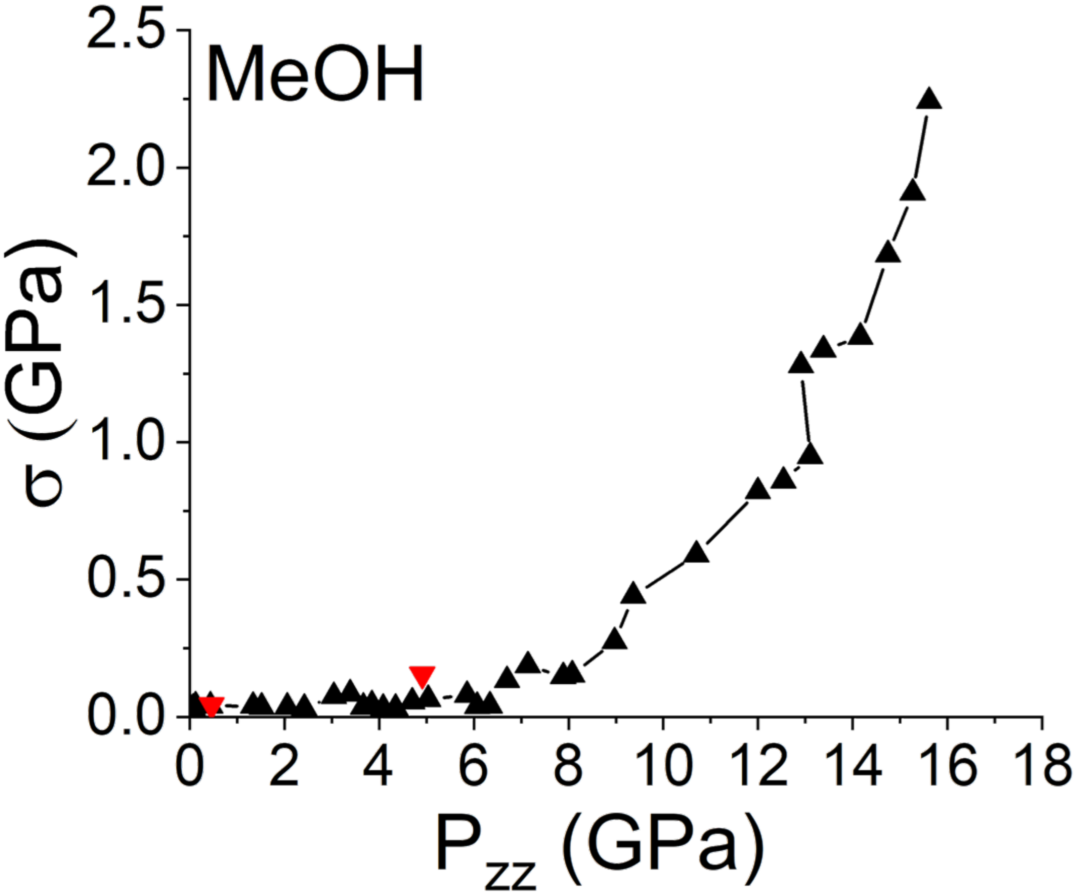

Figure S 13: Hydrostatic limit of MeOH. Black upward triangles represent compression points, red downwards triangles represent decompression points.

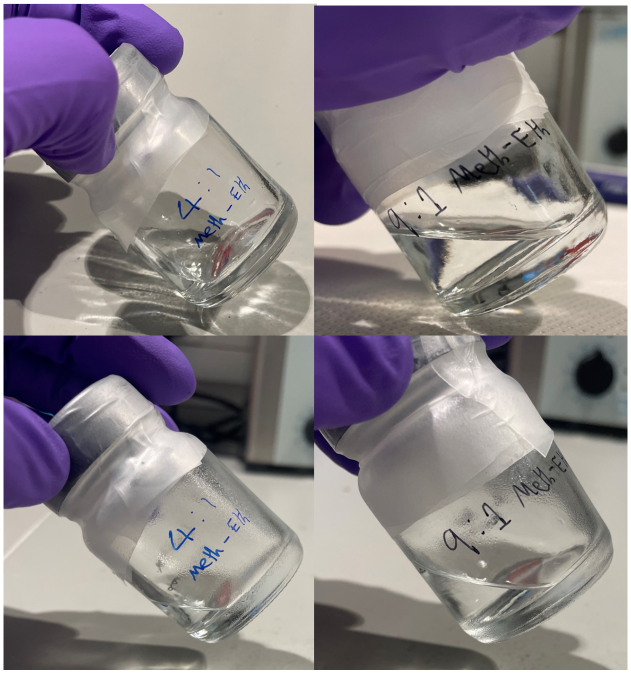

Figure S 14: Stability of 9:1 MeOH–EtOH in a fridge. Left, a mixture of 4:1 MeOH–EtOH before (top) and after (bottom) two weeks storage in a lab fridge. Right, the same photos but for a 9:1 MeOH–EtOH mixture. There is no perceivable difference between the solutions in any of the photos.

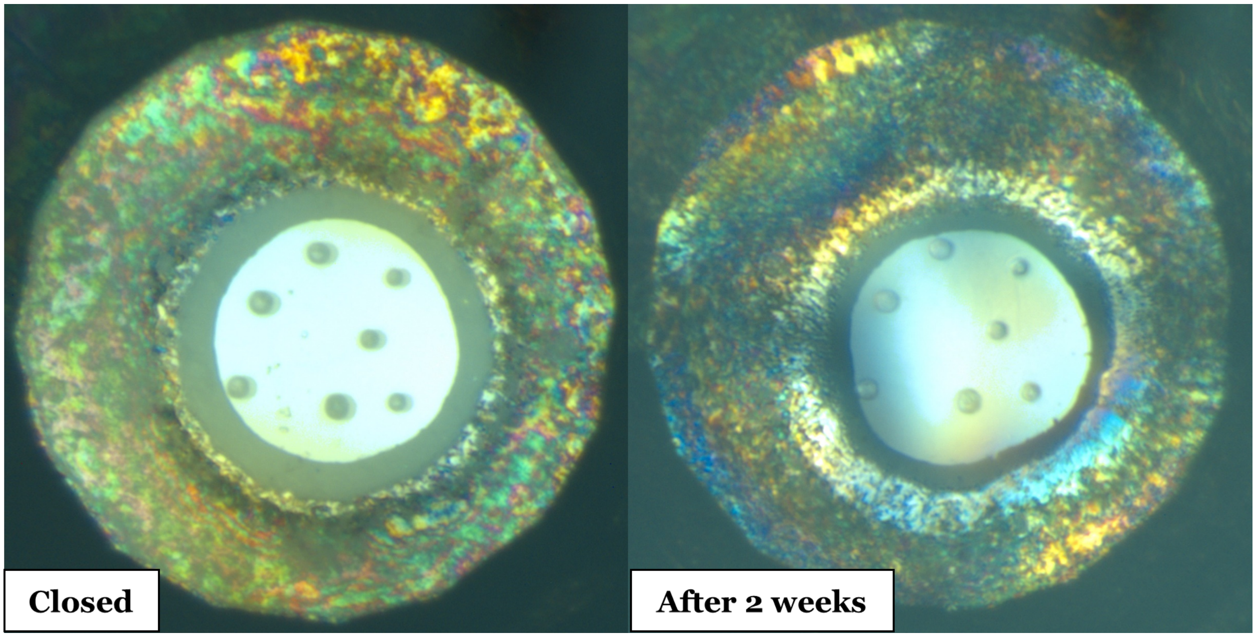

Figure S 15: Stability of 9:1 MeOH–EtOH at pressure. Left, a DAC just closed. Right, the same DAC after 2 weeks above the crystallisation pressure of methanol and ethanol. The PTM has not crystallised. A slight deformation of the gasket is observed as is common for extended experiments at high pressure.

References

Chen, B., Potoff, J. J. & Siepmann, J. I. (2001). *The Journal of Physical Chemistry B*, **105**, 3093–3104.

Developers, T. L., (2025*a*). Lammps manual - compute adf. https://docs.lammps.org/compute_adf.html. Accessed: 2025-07-16.

Developers, T. L., (2025*b*). Lammps manual - compute com/chunk. https://docs.lammps.org/compute_com_chunk.html. Accessed: 2025-07-16.

Developers, T. L., (2025*c*). Lammps manual - compute rdf. https://docs.lammps.org/compute_rdf.html. Accessed: 2025-07-16.

Developers, T. L., (2025*d*). Lammps manual - fix ave/time. https://docs.lammps.org/fix_ave_time.html. Accessed: 2025-07-16.

Developers, T. L., (2025*e*). Lammps manual - fix msd/chunk. https://docs.lammps.org/compute_msd_chunk.html. Accessed: 2025-07-16.

Developers, T. L., (2025*f*). Lammps manual - fix npt. https://docs.lammps.org/fix_nh.html#fix-npt-command. Accessed: 2025-07-16.

Frenkel, D. & Smit, B. (2002). *Understanding Molecular Simulation: from algorithms to applications*. Academic Press.

Gowers, R. J. & Carbone, P. (2015). *The Journal of Chemical Physics*, **142**, 224907–224917.

Group, T. S., (2025). Trappe force field website. http://trappe.oit.umn.edu/. Accessed: 2025-07-16.

Guevara-Carrion, G., Nieto-Draghi, C., Vrabec, J. & Hasse, H. (2008). *The Journal of Physical Chemistry B*, **112**, 16664–16674.

Hoover, W. G. (1985). *Physical Review A*, **31**, 1695–1697.

Im Lab, L. U., (2025). charmm-gui website. https://charmm-gui.org/?doc=input. Accessed: 2025-07-16.

Jo, S., Kim, T., Iyer, V. G. & Im, W. (2008). *Journal of Computational Chemistry*, **29**, 1859–1865.

Jorgensen, W. L., Maxwell, D. S. & Tirado-Rives, J. (1996). *Journal of the American Chemical Society*, **118**, 11225–11236.

Keen, D. A. (2001). *Journal of Applied Crystallography*, **34**, 172–177.

Kim, S., Lee, J., Jo, S., Brooks III, C. L., Lee, H. S. & Im, W. (2017). *Journal of Computational Chemistry*, **38**, 1879–1886.

Klotz, S., Chervin, J. C., Munsch, P. & Le Marchand, G. (2009). *Journal of Physics D: Applied Physics*, **42**, 075413.

Lee, J., Cheng, X., Jo, S., MacKerell, A. D., Klauda, J. B. & Im, W. (2016). *Biophysical journal*, **110**, 641a.

Lee, J., Hitzenberger, M., Rieger, M., Kern, N. R., Zacharias, M. & Im, W. (2020). *The Journal of Chemical Physics*, **153**, 035103–035112.

Motaln, K., Uran, E., Giordano, N., Parsons, S. & Lozinšek, M. (2025). *Journal of Applied Crystallography*, **58(1)**, 221–226.

Schnabel, T., Srivastava, A., Vrabec, J. & Hasse, H. (2007). *The Journal of Physical Chemistry B*, **111**, 9871–9878.

Schnabel, T., Vrabec, J. & Hasse, H. (2005). *Fluid Phase Equilibria*, **233**, 134–143.

SilcsBio, L., (2025). Cgenff website. https://cgenff.com/. Accessed: 2025-07-16.

Thompson, A. P., Aktulga, H. M., Berger, R., Bolintineanu, D. S., Brown, W. M., Crozier, P. S., in't Velt, Pieter J., K. A., Moore, S. G., Nguyen, T. D., Shan, R., Stevens, M. J., Tranchida, J., Trott, C. & J., P. S. (2022). *Computer Physics Communications*, **271**, 108171–108205.

Vanommeslaedhe, K., Hatcher, E., Acharya, C., Kundu, S., Zhong, J., Shim, J., Darian, E., Guvench, O., Lopes, P., Vorobyov, I. & Mackerell Jr, A. D. (2010). *Journal of Computational Chemistry*, **31**, 671–690.

Zenodo, (2025). data. https://doi.org/10.5281/zenodo.16028744/. Accessed: 2025-07-17.
